# The anti-breast cancer stem cell properties of gold(i)-non-steroidal anti-inflammatory drug complexes†

**DOI:** 10.1039/d2sc04707a

**Published:** 2022-12-12

**Authors:** Alice Johnson, Chibuzor Olelewe, Jong Hyun Kim, Joshua Northcote-Smith, R. Tyler Mertens, Ginevra Passeri, Kuldip Singh, Samuel G. Awuah, Kogularamanan Suntharalingam

**Affiliations:** a School of Chemistry, University of Leicester Leicester UK k.suntharalingam@leicester.ac.uk; b Biomolecular Sciences Research Centre, Sheffield Hallam University Sheffield UK alice.johnson@shu.ac.uk; c Department of Chemistry, University of Kentucky Lexington Kentucky USA awuah@uky.edu; d Department of Pharmaceutical Sciences, University of Kentucky Lexington Kentucky USA

## Abstract

The anti-breast cancer stem cell (CSC) properties of a series of gold(i) complexes comprising various non-steroidal anti-inflammatory drugs (NSAIDs) and triphenylphosphine 1–8 are reported. The most effective gold(i)-NSAID complex 1, containing indomethacin, exhibits greater potency for breast CSCs than bulk breast cancer cells (up to 80-fold). Furthermore, 1 reduces mammosphere viability to a better extent than a panel of clinically used breast cancer drugs and salinomycin, an established anti-breast CSC agent. Mechanistic studies suggest 1-induced breast CSC death results from breast CSC entry, cytoplasm localisation, an increase in intracellular reactive oxygen species levels, cyclooxygenase-2 downregulation and inhibition, and apoptosis. Remarkably, 1 also significantly inhibits tumour growth in a murine metastatic triple-negative breast cancer model. To the best of our knowledge, 1 is the first gold complex of any geometry or oxidation state to demonstrate anti-breast CSC properties.

## Abstract 2

The anti-breast cancer stem cell (CSC) properties of a series of gold(i) complexes comprising various non-steroidal anti-inflammatory drugs (NSAIDs) and triphenylphosphine 1–8 are reported.

## Graphical abstract

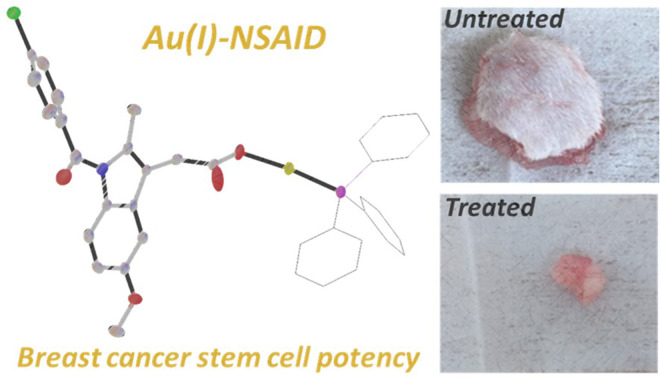

## Introduction

Breast cancer is the most common cancer for women worldwide, with 2.3 million cases and 685 000 fatalities (around 1876 people per day) recorded yearly according to the latest WHO statistics.^1^ Current breast cancer therapies are unable to positively impact the lives of a significant proportion of diagnosed patients (24% of all breast cancer patients are expected to die 10 years post diagnosis).^2^ Breast cancer recurrence is strongly linked to the existence of breast cancer stem cells (CSCs), a sub-population of breast cancer cells that have the ability to self-renew, differentiate, and form secondary tumours.^3,4^ Basal-like, claudin-low, and Her2-positive breast tumours are associated with the lowest life expectancy and display the largest proportions of breast CSCs.^5^ Breast CSCs are also thought to play an important role in metastasis, indeed, clinical studies have found much greater proportions of breast CSC-like cells in metastatic tumours compared to the primary site.^6–8^ Breast CSCs divide slower than bulk breast cancer cells and thus can overcome conventional chemotherapeutics and radiation regimens, which tend to target fast growing cells.^9–12^ The very low proportion of breast CSCs within a given tumour site and their tendency to reside in hard to reach niches, means that they can be missed by surgery as well. After surviving treatment, breast CSCs are believed to be able to regenerate tumours in the original site or produce invasive breast cancer cells that can colonise distant organs.^13^ The clinical implication of breast CSCs means that treatments must have the ability to remove heterogeneous breast cancer populations in their entirety, including breast CSCs, otherwise breast CSC-mediated relapse could occur. Potential breast CSC therapeutic targets such as cell surface markers, dysregulated signaling pathways, and components within the microenvironments in which they reside have been identified,^6^ however almost 20 years since the discovery of breast CSCs, there is still no clinically approved drug that can completely remove breast CSCs at their clinically administered dose(s).^14^

Small molecule chemotherapeutic strategies employed to treat non-metastasised and metastasised breast cancer are largely reliant on anthracyclines, taxanes, nucleobase-like compounds, natural product derivatives, and platinum(ii)-based agents (such as cisplatin and carboplatin).^15^ All of these drug options are unable to remove breast CSCs at their clinically administered doses.^16^ The use of platinum(ii) complexes in breast cancer treatment regimens has motivated several studies on the development of isoelectronic gold(iii) complexes as alternative drug candidates.^17–22^ Most gold(iii) complexes suffer from thiol-mediated reduction within biological systems, to the corresponding gold(i) congener and/or metallic gold.^23^ Cyclometalated gold(iii) complexes with multidentate ligands (containing deprotonated C-donor atoms) are resistant to reduction under physiological conditions.^23–27^ Despite the large body of work on anticancer gold(iii) complexes no study has looked into their anti-breast CSC properties,^28^ and only one study has looked into their potency toward CSC-like populations within other tissue types.^29^ A gold(iii) *meso*-tetraphenylporphyrin complex possessing high stability in the presence of glutathione and serum albumin, and strong *in vitro* and *in vivo* (murine models) activity against a range of bulk cancer cells, was reported to inhibit spheroid formation from single cell suspensions of CSC-rich U-87 MG glioblastoma cells at micromolar and nanomolar concentrations.^29^ The gold(iii) complex induced toxicity by reducing NANOG (a stemness marker) expression and downregulating 16 microRNAs linked to glioblastoma stem cell function.^29^

The use of gold(i) complexes in medicine is more prevalent than gold(iii) complexes owing to the clinical application of gold(i) salts as anti-rheumatoid arthritis agents.^23,30–34^ Although there are now a plethora of studies on the anti-bulk cancer cell properties of structurally diverse gold(i) complexes, only a handful of gold(i) complexes have been reported to effectively reduce CSC viability.^35–38^ A series of binuclear gold(i) complexes containing mixed bridging bis(*N*-heterocyclic carbene) and diphosphine ligands were identified to disrupt spheroid formation from single cell suspensions of CSC-rich U-87 MG glioblastoma and HeLa cervical carcinoma cells at low micromolar concentrations.^35^ Mononuclear gold(i) complexes with bulky phosphine and halide ligands inhibited the growth of HeLa cervical carcinoma spheroids to a reasonable level at micromolar concentrations.^36^ Both classes of gold(i) complexes are thought to effect toxicity through covalent interactions with thiol-containing proteins.^35,36^ A gold(i) complex with a derivatised phosphaphenalene ligand and a thio-sugar suppressed CSC-rich NCH421k, NCH644, and NCH660h glioblastoma cell proliferation at micromolar concentrations.^37^ The exact mechanism of action of this complex was not reported but it was shown to induce apoptosis.^37^ The anti-rheumatoid arthritis drug auranofin was reported to deplete stem cell-like lung cancer cell side populations at micromolar concentrations and impair their tumorigenicity in a xenograft mouse model.^38^ Auranofin was characterised to induce cell toxicity by increasing intracellular reactive oxygen species (ROS) levels and depleting cellular ATP concentrations (by disrupting glycolysis).^38^

To date there have been no reports on the anti-breast CSC properties of gold(i) complexes.^28^ Inspired by the promising, yet underexplored, anti-CSC properties of gold complexes, we sought to prepare gold(i) complexes containing a stabilising phosphine ligand and a panel of non-steroidal anti-inflammatory drugs (NSAID) and determine their anti-breast CSCs properties. NSAIDs are inhibitors of cyclooxygenase-2 (COX-2), an enzyme that is overexpressed in mammary carcinomas (with CSC-enriched populations) and associated to breast cancer progression.^39,40^ It should be noted that several metal-NSAID complexes have been previously reported, and their binding to biomolecules such as DNA and HSA has been well characterised using spectroscopic methods.^41–45^ Many of the metal-NSAID complexes display anti-inflammatory, antibacterial, and antiproliferative properties.^46^ Encouragingly, some metal-NSAID complexes exhibit greater cytotoxicity toward breast cancer cells than cisplatin.^46^ We have used NSAIDs in combination with endogenous metals (copper, manganese, and zinc) to potently and selectively kill breast CSCs over other cell types.^47–49^ To our surprise, the combination of gold and unmodified NSAIDs within a single chemical entity has not been reported. Here we investigate this knowledge space in the context of breast CSC activity.

## Results and discussion

### Synthesis and characterisation of gold(i)-non-steroidal anti-inflammatory drug complexes

**Scheme 1:**
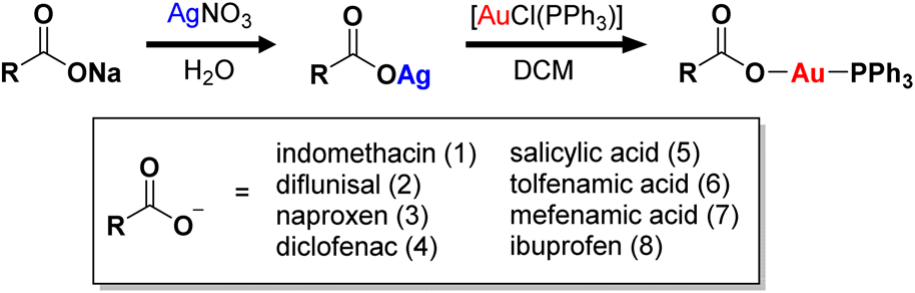
Reaction scheme for the preparation of the gold(i) complexes containing triphenylphosphine and indomethacin, diflunisal, naproxen, diclofenac, salicylic acid, tolfenamic acid, mefenamic acid, or ibuprofen (1–8).

**Fig. 1:**
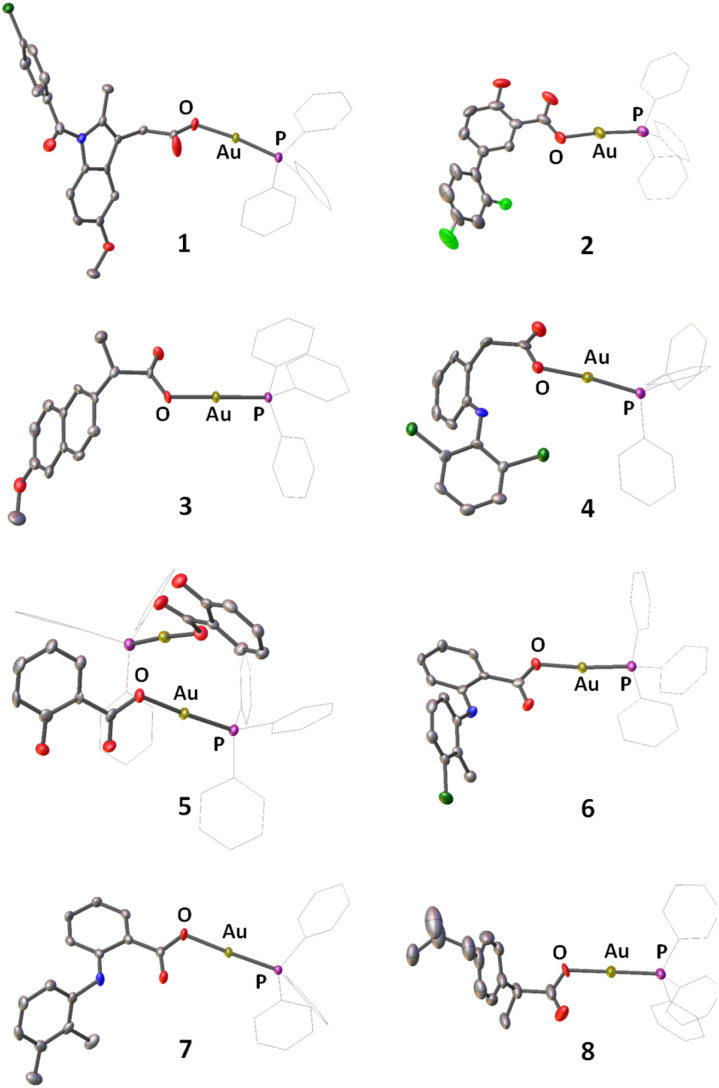
X-ray structures of 1–8 comprising of triphenylphosphine and indomethacin, diflunisal, naproxen, diclofenac, salicylic acid, tolfenamic acid, mefenamic acid, or ibuprofen. Thermal ellipsoids are drawn at 50% probability. C atoms are shown in grey, N in dark blue, O in red, P in purple, Cl in dark green, F in light green, and Au in gold. The H atoms have been omitted and the Ph groups in PPh_3_ are represented as wireframes for clarity.

A series of gold(i) complexes were prepared with triphenylphosphine and various NSAIDs (1–8) and their chemical structures are depicted in Scheme 1 and Fig. 1. The sodium salts of the NSAIDs (indomethacin, diflunisal, naproxen, diclofenac, salicylic acid, tolfenamic acid, mefenamic acid, or ibuprofen) were reacted with one equivalent of silver nitrate in water (in the dark) to form the corresponding silver(i)-NSAID complex. The silver(i)-NSAID complexes were then directly reacted with one equivalent of chloro(triphenylphosphine)gold(i) in dichloromethane (in the dark) to yield the gold(i)-NSAID complexes 1–8 as white solids in reasonable yields (35–79%). The gold(i)-NSAID complexes 1–8 were fully characterised by ^1^H, ^31^P (and ^19^F) NMR and infra-red spectroscopy, elemental analysis, and single crystal X-ray crystallography (Fig. S1–S18 and Tables S1–S3, see ESI†). Retention of the triphenylphosphine-gold(i) coordination in 1–8 was confirmed by the relative downfield chemical shift of the ^31^P NMR signals corresponding to 1–8 (27.37–27.67 ppm) compared to free triphenylphosphine, (−5.55 ppm, Fig. S19†). The difference between the vibrational stretching frequencies between the asymmetric, *ν*_asym_(CO_2_) and symmetric, *ν*_sym_(CO_2_) carboxylato peaks gives an indication of the binding mode of the associated carboxylic acid group to a given metal centre.^50^ According to the IR spectra, the difference (*Δ*) between *ν*_asym_(CO_2_) and *ν*_sym_(CO_2_) stretching bands for 1–8 varied between 196–241 cm^−1^ (Fig. S18†), suggestive of a monodentate coordination mode for the carboxylate group on the NSAIDs to the gold(i) centre. Purity of the bulk solids of 1–8 was confirmed by elemental analysis (see ESI†). Single crystals of 1–8 suitable for X-ray diffraction studies were obtained by layer-diffusion of hexane into a DCM solution of 1–8 (CCDC 2175736–2175743, Fig. 1 and Tables S1 and S2†). Selected bond distances and bond angles data are presented in Table S3.† The structures of 1–8 all consist of gold(i) bound to triphenylphosphine and the corresponding NSAID *via* the hydroxyl oxygen within the carboxylate group. The gold(i) coordination sphere is consistent with the aforementioned spectroscopic and analytic data for 1–8. The P_(triphenylphosphine)_–Au–O_(NSAID)_ angle varied from 173.2° to 177.7° suggesting that 1–8 adopt pseudo linear structures. The average Au–O (2.06 Å) and Au–P (2.21 Å) bond distances are consistent with bond parameters for related complexes.^51–54^

The lipophilicity of 1–8 was determined by measuring the extent to which it partitioned between octanol and water, *P*. The experimentally determined log *P* values varied from 0.71 ± 0.04 to 1.64 ± 0.42 (Table S4†). The log *P* values for 1–8 suggest that the complexes should be readily taken up by cells and be adequately soluble in aqueous solutions. The stability of 1–8 in solutions relevant for cell-based studies was determined by UV-vis spectroscopy studies. In DMSO, the UV-vis trace for 1–5 (50 μM) remained largely unaltered over the course of 24 h at 37 °C suggestive of stability (Fig. S20†). In contrast, the absorption bands associated to 6–8 (50 μM) changed dramatically under the same conditions, suggestive of instability (Fig. S20†). Further studies with the DMSO-stable complexes 1–5 (50 μM) revealed that 1–4, but not 5, was stable in PBS : DMSO (1 : 1) over the course of 24 h at 37 °C (Fig. S21†). The DMSO- and PBS-stable complexes 1–4 (50 μM) were also deemed stable in MEGM : DMSO (1 : 1) over the course of 24 h at 37 °C (Fig. S22†). Time course ^31^P{^1^H} and ^1^H NMR spectroscopy studies were carried out to confirm the solution stability of 1–4. The ^31^P{^1^H} NMR spectra for 1–3 (10 mM) in DMSO-*d*_6_ displayed a single signal throughout the course of 72 h corresponding to the intact complexes (Fig. S23–S25†). The ^1^H NMR spectra for 1–3 (10 mM) remained unchanged over the same period (Fig. S26–S28†). In contrast, the ^31^P{^1^H} and ^1^H NMR spectra for 4 in DMSO-*d*_6_ displayed distinct changes over the course of 72 h (Fig. S29–S30†). In the ^31^P{^1^H} NMR spectrum, the signal for intact 4 (at 27.05 ppm) completely disappeared after 48 h and was replaced by a signal corresponding to triphenylphosphine oxide (at 25.47 ppm) (Fig. S29†). The formation of triphenylphosphine oxide was also detected in the ^1^H NMR spectrum (Fig. S30†). Additionally, plating of elemental gold was observed over the course of 72 h. This suggests that 4 is unstable in solution, and more specifically that the gold(i) centre in 4 undergoes reduction to gold(0) which precipitates out of solution and the triphenylphosphine ligand undergoes oxidation to triphenylphosphine oxide which remains in solution. Taken together the UV-vis and NMR spectroscopy studies suggest that out of the eight gold(i)-NSAID complexes prepared, 1–3 are stable in solution and thus suitable for cell-based studies.

In light of the stability data, we looked more closely at the Au–P bond distances and the ^31^P{^1^H} NMR chemical shifts of 1–8 in order to explain their varying stabilities. The average Au–P bond distance for 1–3 (2.2093 Å) is slightly shorter than the average Au–P bond distance for 4–8 (2.2143 Å) (Table S3†). The difference in the Au–P bond distances is reflected in the ^31^P{^1^H} NMR chemical shifts (Fig. S2, S4, S7, S9, S11, S13, S15 and S17†). The average ^31^P{^1^H} NMR chemical shift for 1–3 is 27.44 ppm whereas for 4–8 it is 27.56 ppm. Therefore, the varying stabilities of 1–3 and 4–8 could be, in part, related to the strength of their respective Au–P bond.

### Breast cancer stem cell and bulk breast cancer cell potency

The potency of 1–3 against a panel of metastasis-prone human and murine bulk breast cancer cells (MDA-MB-231, MDA-MB-468, HMLER, and 4T1) and breast CSC-enriched cells (HMLER-shEcad) was determined using the MTT assay. The IC_50_ values were determined from dose–response curves (Fig. S31–S36†) and are summarised in Tables 1 and S5.† The gold(i)-NSAID complexes 1–3 exhibited micromolar or sub-micromolar toxicities towards MDA-MB-231, MDA-MB-468, HMLER, and 4T1 cells with the indomethacin-bearing complex 1 displaying the highest overall potency. Remarkably, 1–3 exhibited sub-micromolar or nanomolar toxicities towards HMLER-shEcad cells, again with the indomethacin-containing complex 1 displaying the highest potency. Notably, 1 exhibited 3- to 80-fold greater potency (*p* < 0.05, *n* = 18) for breast CSC-enriched HMLER-shEcad cells over the panel of bulk breast cancer cells tested (Tables 1 and S5†). The breast CSC potency of 1 was significantly greater (*p* < 0.05, *n* = 18) than a series of clinically approved breast cancer drugs (5-fluorouracil, capecitabine, cisplatin, and carboplatin), a clinically tested anti-breast CSC agent (salinomycin), and any previously reported metal-containing agent (under identical conditions) (Table 1 and Fig. S37 andS38†).^47,48,55^ As a measure of therapeutic potential, the cytotoxicity of 1 towards embryonic kidney HEK 293 cells was determined. The complex, 1 was 34-fold less potent toward HEK 293 cells (IC_50_ value = 1.92 ± 0.23 μM, Fig. S39†) than HMLER-shEcad cells, indicating selective toxicity for breast CSCs over non-tumorigenic cells.

**Table 1:** IC_50_ values of the gold(i)-NSAID complexes, 1–3, 5-fluorouracil, capecitabine, cisplatin, carboplatin, and salinomycin against HMLER cells, HMLER-shEcad cells, and HMLER-shEcad mammospheres

Compound	HMLER IC_50_/nM	HMLER-shEcad IC_50_/nM	Mammosphere IC_50_/μM
1	190 ± 10	56 ± 1	2 ± 0.1
2	221 ± 3	138 ± 7	8 ± 1
3	183 ± 1	63 ± 6	8 ± 1
5-Fluorouracil	41 050 ± 5303	49 100 ± 5940	15 ± 1
Capecitabine	>100 000	>100 000	>133
Cisplatin^a^	2565 ± 21	5645 ± 304	14 ± 2
Carboplatin^a^	67 310 ± 2800	72 390 ± 7990	18 ± 1
Salinomycin^a^	11 430 ± 420	4230 ± 350	19 ± 2

a Reported in ref. 47, 55, and 57.

Control cytotoxicity studies with chloro(triphenylphosphine)gold(i) and indomethacin (the NSAID present in 1) individually and combined were also conducted. Indomethacin was non-toxic towards HMLER and HMLER-shEcad cells within the concentration range tested (IC_50_ > 100 μM) (Fig. S40 and Table S6†). Chloro(triphenylphosphine)gold(i) was up to 3.8-fold (*p* < 0.05) less toxic towards HMLER and HMLER-shEcad cells than 1 (Fig. S41 and Table S6†). When dosed as a 1 : 1 mixture, the combined treatment of indomethacin and chloro(triphenylphosphine)gold(i) showed a significant (*p* < 0.05) reduction in potency towards HMLER and HMLER-shEcad cells compared to 1 (Fig. S42 and Table S6†). Overall, this demonstrates that 1 is significantly better at killing bulk breast cancer cells and breast CSCs than chloro(triphenylphosphine)gold(i) or indomethacin alone or when treated together. Further control cytotoxicity studies with the gold(i) anti-rheumatoid arthritis agent, auranofin were performed. Auranofin was 2-fold (*p* < 0.05) more potent towards HMLER cells than HMLER-shEcad cells (Fig. S43 and Table S6†). Therefore, auranofin is not selective for breast CSCs over bulk breast cancer cells unlike the gold(i)-NSAID complexes 1–3.

**Fig. 2:**
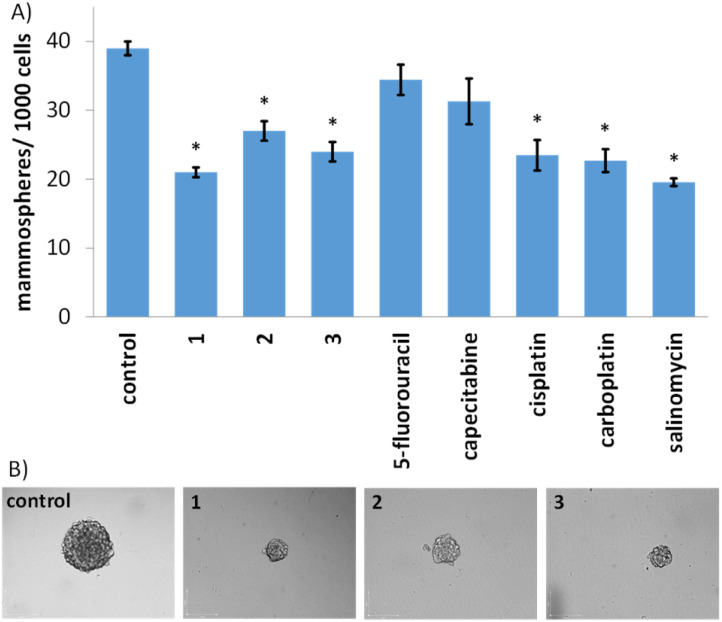
(A) Quantification of mammosphere formation with HMLER-shEcad cells untreated and treated with 1–3, 5-fluorouracil, capecitabine, cisplatin, carboplatin, and salinomycin at their respective IC_20_ values after 5 days incubation. Error bars = SD and Student's *t*-test, * = *p* < 0.05. (B) Representative bright-field images (×10) of the mammospheres in the absence and presence of 1–3 at their respective IC_20_ values after 5 days incubation.

Given the impressive cytotoxicity of 1–3 towards breast CSCs grown in monolayer cultures, their activity towards three-dimensional mammospheres was determined. Mammospheres are more representative of solid tumours compared to monolayer cultures and provide a reliable readout of *in vivo* potential.^56^ The addition of 1–3 (at their IC_20_ value) to single cell suspensions of HMLER-shEcad cells markedly reduced the number and size of mammospheres formed after 5 days incubation (Fig. 2A and B). The greatest inhibitory effect was observed for 1 and it was comparable or better than the effect observed for any of the clinically approved breast cancer drugs tested (5-fluorouracil, capecitabine, cisplatin, and carboplatin) and salinomycin (under identical treatment conditions) (Fig. 2A and B and S44†). To determine the effect of 1–3 on mammosphere viability, the colorimetric resazurin-based reagent, TOX8 was used. All of the complexes displayed micromolar potency (Table 1 and Fig. S45†). Notably, 1 displayed up to 4-fold greater potency for mammospheres than 2 or 3, and up to 9-fold greater potency than the clinically approved breast cancer drugs tested or salinomycin (Table 1 and Fig. S46†).^55,57^ Collectively, the mammosphere studies show that the gold(i)-NSAID complexes, especially 1, are able to reduce breast CSC mammosphere formation, size, and viability.

### Cellular mechanism of action

Cellular uptake studies were carried out to determine the breast CSC permeability of 1–3. HMLER-shEcad cells were incubated with 1–3 (0.25 μM for 24 h) and the intracellular gold content was determined by inductively coupled plasma mass spectrometry (ICP-MS) (Fig. S47†). All three gold(i)-NSAID complexes were readily taken up by HMLER-shEcad cells with whole cell uptake ranging from 31 ng of Au/10^6^ cells for 3 to 46 ng of Au/10^6^ for 2. Fractionation studies were carried out with the most effective gold(i)-NSAID complex 1. HMLER-shEcad cells were incubated with 1 (0.25 μM for 24 h), harvested, and fractionated to determine the localisation of 1 within breast CSCs. Significant amounts of internalised gold were detected in the cytoplasm (80%) with the remainder detected in the nucleus and cell membrane (Fig. S48†). This implies that the intracellular target for 1 in breast CSCs is likely to be biomolecules within the cytoplasm.

**Fig. 3:**
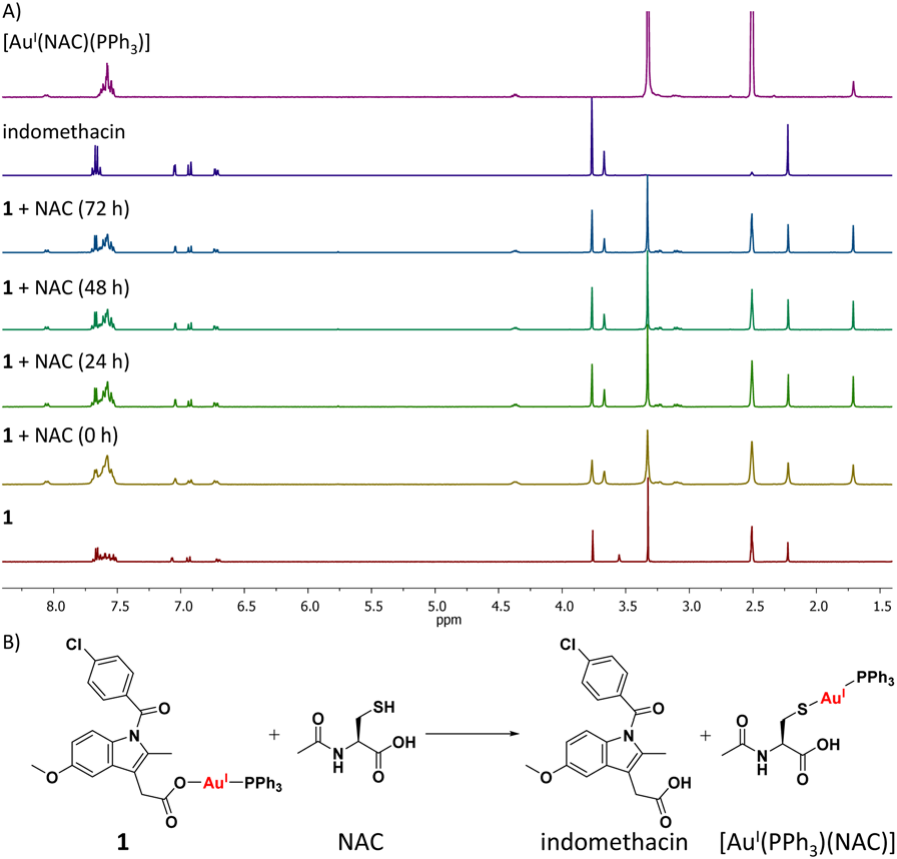
(A) ^1^H NMR spectra for complex 1 (10 mM) in DMSO-*d*_6_, in the absence and presence of *N*-acetylcysteine (NAC, 10 mM) over the course of 72 h at 37 °C. The ^1^H NMR spectra of indomethacin and [Au^I^(NAC)(PPh_3_)] (both 10 mM) in DMSO-*d*_6_ are also provided. (B) Representative scheme for the reaction of 1 with NAC.

As the mechanism of toxicity of many gold(i) complexes is associated to their interaction with thiol groups in proteins,^23^ the reaction of 1 with *N*-acetylcysteine (NAC) and glutathione (GSH), model thiol-containing biomolecules, was probed using ^1^H and ^31^P{^1^H} NMR spectroscopy studies (over 72 h at 37 °C). DMSO-*d*_6_ was used to ensure that the reactants and products remain in solution at the relatively high concentrations (millimolar concentration) required to obtain reliable NMR spectra. ^1^H NMR studies in DMSO-*d*_6_ revealed that the addition of 1 (10 mM) to a stoichiometric amount of NAC or GSH yielded free indomethacin and [Au^I^(NAC)(PPh_3_)] or [Au^I^(GSH)(PPh_3_)], respectively (Fig. 3A and S49†). The reaction occurred immediately and the products remained unchanged for 72 h. The ^31^P{^1^H} NMR studies also indicated the immediate formation of [Au^I^(NAC)(PPh_3_)] or [Au^I^(GSH)(PPh_3_)] (Fig. S50–S51†). [Au^I^(NAC)(PPh_3_)] and [Au^I^(GSH)(PPh_3_)] were independently prepared *in situ* by reacting [Au^I^(acac)(PPh_3_)]^58^ with NAC or GSH in DMSO-*d*_6_, to confirm the abovementioned assignments. These studies suggest that 1 is able to interact with thiol-containing biomolecules and release indomethacin (Fig. 3B and Scheme S1†).

**Fig. 4:**
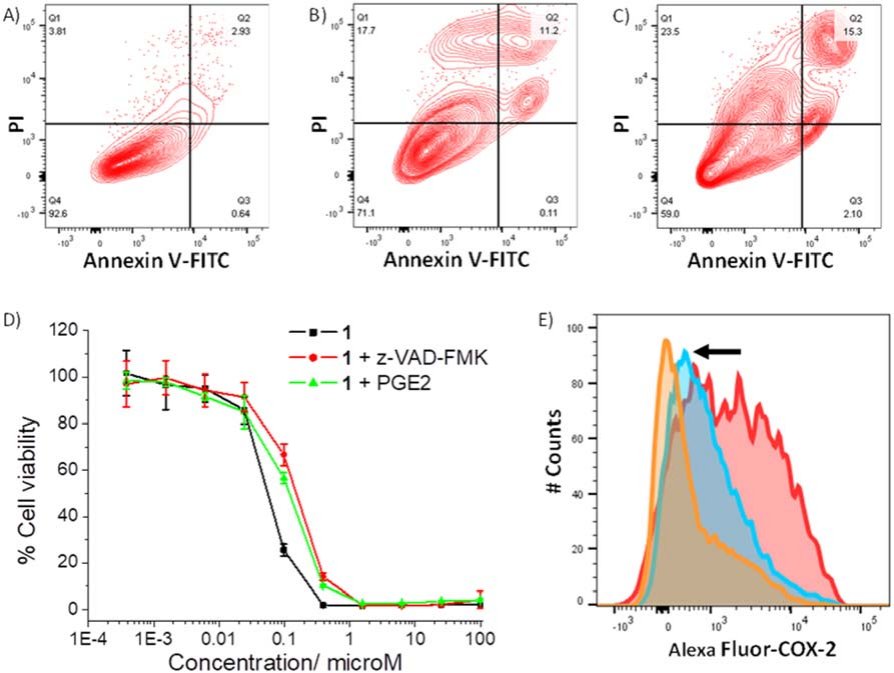
(A–C) FITC Annexin V-propidium iodide binding assay plots of untreated HMLER-shEcad cells, and HMLER-shEcad cells treated with 1 (IC_50_ value ×2 for 48 h) or cisplatin (25 μM for 48 h). (D) Representative dose–response curves for the treatment of HMLER-shEcad cells with 1 in the absence and presence of z-VAD-FMK (5 μM) or PGE2 (20 μM), after 72 h incubation. (E) Representative histograms displaying the green fluorescence emitted by anti-COX-2 Alexa Fluor 488 nm antibody-stained HMLER-shEcad cells treated with LPS (2.5 μM) for 24 h followed by 48 h in fresh media (red) or media containing 1 (IC_50_ value, blue) or indomethacin (20 μM, orange).

As 1 readily reacts with GSH (Fig. S49 and S51†) and accumulates in the cytoplasm of breast CSCs (Fig. S48†) where GSH is predominately localised, 1 could potentially perturb the GSH redox buffering system in breast CSCs and induce intracellular ROS elevation.^59^ The ability of 1 to perturb ROS levels in HMLER-shEcad cells over a 24 h period was determined using 2′,7′-dichlorodihydro-fluorescein diacetate (DCFH-DA), a well-established ROS indicator, and flow cytometry. HMLER-shEcad cells treated with 1 (0.4 μM) exhibited a time-dependent increase in intracellular ROS levels, peaking at 6 h exposure (104% increase, *p* < 0.05) (Fig. S52–S53†). Prolonged (16–24 h) exposure of 1 led to statistically insignificant increases in ROS levels (*p* > 0.05). An increase in intracellular ROS levels, as observed after the treatment of HMLER-shEcad cells with 1, can prompt apoptosis.^60^ Apoptosis induces morphological changes that can lead to cell membrane rearrangement. This process results in the translocation of phosphatidylserine residues to the membrane exterior, which can be detected by Annexin V.^61^ Damaged cell membranes also facilitate propidium iodide uptake. Using a dual FITC annexin V-propidium iodide staining flow cytometry assay, we explored the occurrence of apoptosis in HMLER-shEcad cells treated with 1. Dosage with 1 (IC_50_ value ×2) over a long incubation period (48 h) induced large populations of cells to undergo late-stage apoptosis (Fig. 4A and B). This was comparable to dosage with cisplatin (25 μM for 48 h), a well-known apoptosis inducer (Fig. 4C). To further corroborate the occurrence of 1-mediated apoptosis, cytotoxicity studies were carried out in the presence of z-VAD-FMK (5 μM, 72 h), a peptide-based caspase-dependent apoptosis inhibitor.^62^ The IC_50_ value of 1 towards HMLER-shEcad cells increased significantly in the presence of z-VAD-FMK (IC_50_ value = 152 ± 7 nM, *p* < 0.05, Fig. 4D) further confirming that 1 induces apoptosis in breast CSCs.

As the gold(i)-NSAID complex 1 releases indomethacin upon reaction with thiol-containing biomolecules, we investigated whether the mechanism of action of 1 involved COX-2 downregulation and inhibition. HMLER-shEcad cells pre-treated with lipopolysaccharide (LPS) (2.5 μM for 24 h), to increase basal COX-2 levels, were treated with 1 (IC_50_ value for 48 h) or indomethacin (20 μM for 48 h), and the COX-2 expression was determined by flow cytometry. COX-2 expression decreased upon treatment with 1 and indomethacin, suggesting that the cytotoxic mechanism of action of 1 is related to COX-2 downregulation (Fig. 4E). To determine if 1 evokes COX-2-dependent breast CSC death, cytotoxicity studies were performed with HMLER-shEcad cells in the presence of prostaglandin E2 (PGE2) (20 μM, 72 h), the product of COX-2-mediated arachidonic acid metabolism.^63^ The potency of 1 towards HMLER-shEcad cells decreased significantly in the presence of PGE2 (IC_50_ value = 116 ± 9 nM, *p* < 0.05, Fig. 4D), suggesting that 1 induces COX-2-dependent breast CSC death. The COX-2 inhibitory properties of 1, indomethacin, and chloro(triphenylphosphine)gold(i) were investigated using an enzyme immunoassay. The IC_50_ values, the concentration required to inhibit COX-2-catalysed conversion of arachidonic acid to prostaglandin by 50%, are reported in Table S7.† The gold(i)-NSAID complex 1 inhibited COX-2 activity in a concentration dependent manner, to a similar extent to indomethacin (Fig. S54†). This shows that despite the coordination of indomethacin to gold in 1, its COX-2 inhibitory effect is retained. COX-2 dosed with chloro(triphenylphosphine)gold(i) (up to 250 μM) largely maintained its ability to convert arachidonic acid to prostaglandin, suggestive of limited inhibition (Fig. S54†). Collectively, the flow cytometry, cytotoxicity, and enzyme immunoassay studies show that 1 not only downregulates COX-2 expression in breast CSCs but also directly inhibits COX-2 activity, and that this is pertinent to 1-induced breast CSC death.

The intracellular redox state in breast CSCs is very finely controlled and balanced, therefore, perturbation of the ROS balance can lead to selective CSC toxicity.^64,65^ COX-2 is overexpressed in breast CSCs and plays a functional role in their proliferation,^66,67^ therefore COX-2 downregulation or inhibition is an effective way of sensitising breast CSCs to cytotoxic agents such as ROS-inducing metal complexes. Taken together, our mechanistic data shows that the gold(i)-NSAID complex 1 increases intracellular ROS levels and reduces COX-2 expression and activity in breast CSCs. This may be the underlying reason for the selective potency observed for 1 towards breast CSCs over bulk breast cancer cells (Tables 1 and S5†). ROS elevation most likely occurs courtesy of the interaction of 1 (*via* the gold(i) moiety) with GSH and consequent perturbation of the GSH redox buffering system. COX-2 downregulation and inhibition occurs as a result of the indomethacin moiety present in 1. Indomethacin has been reported to inhibit COX-2 activity and downregulate COX-2 expression.^68^

### Antitumour efficacy in a murine breast cancer model

**Fig. 5:**
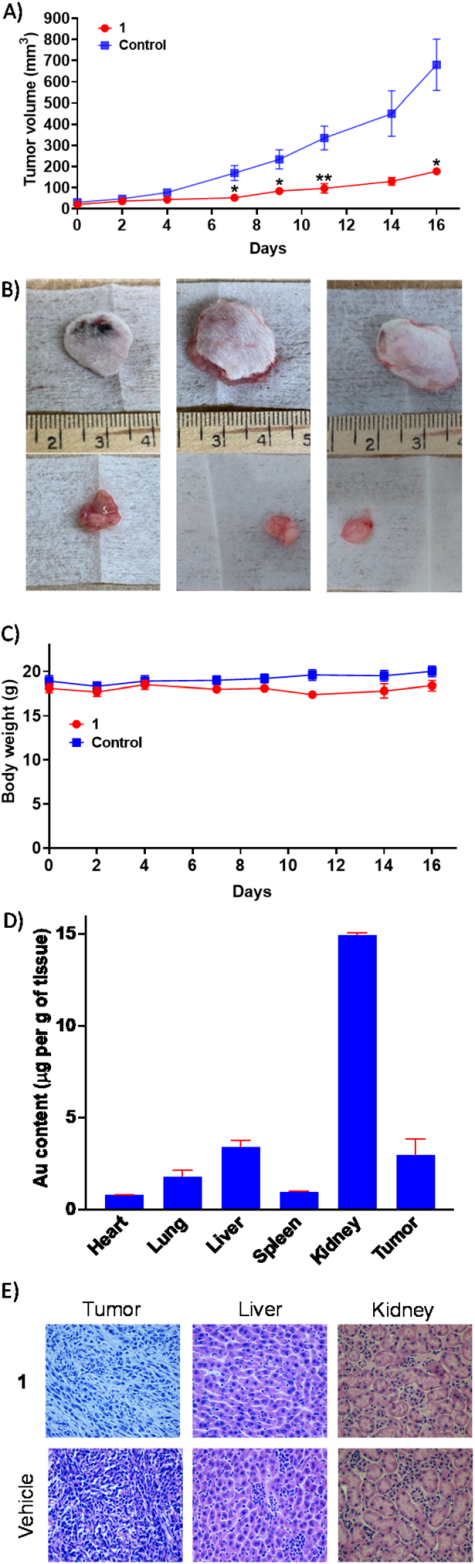
(A) The change in tumour volume of 4T1-bearing mice (1 million cells inoculated, *n* = 5) over 16 days, following intraperitoneal administration of 1 or the vehicle three times a week. Unpaired *t*-test, *p < 0.05 and **p < 0.01. (B) Representative images of excised tumours from 1- or vehicle-treated mice. (C) The change in body weight of 1- or vehicle-treated mice during the efficacy study lasting 16 days. (D) Biodistribution of gold in 4T1-bearing mice following 1 treatment. (E) Hematoxylin and eosin (H&E) staining indicates reduced cellularity in tumour tissue obtained from 1-treated mice (compared to vehicle-treated mice), and unaltered cellularity in liver and kidney tissue obtained from 1-treated mice (compared to vehicle-treated mice).

The most active gold(i)-NSAID complex 1 was evaluated in an immunocompetent breast cancer mouse model to gauge *in vivo* antitumour efficacy. Specifically, a murine 4T1 triple-negative breast cancer model was used and 1 was administered intraperitoneally three times a week at a dose of 10 mg kg^−1^ (*n* = 5 mice). An independent control group (*n* = 5 mice) was treated with the vehicle at the same time points *via* the same administration route. The 1-treated group displayed significant tumour growth inhibition (Fig. 5A and B) and maintained their body weight (Fig. 5C) relative to the control group throughout the course of the study. This implies that 1 is a relatively safe and efficacious antiproliferative agent. Gold biodistribution studies demonstrated that a substantial amount of 1 was found in tumour tissue (3 μg g^−1^ of tissue, Fig. 5D). Notably, the gold content in the kidney (15 μg g^−1^ of tissue) was 4-19-fold larger than the heart, lung, liver, or spleen (Fig. 5D). The comparatively large amount of gold detected in the kidneys could be due to this organ being a major clearance depot for 1. Subsequently, we assessed histological changes in tumour, liver, and kidney tissue. Reduced cellularity was observed in tumour tissue obtained from the 1-treated group compared to the control group, indicative of promising *in vivo* potency (Fig. 5E). Reassuringly there was no alteration to cellularity in the kidney or liver tissue obtained from the 1-treated group compared to the control group (Fig. 5E). Overall, the *in vivo* studies clearly show that 1 is able to effectively reduce breast tumour growth in a murine model without inducing significant systemic toxicity.

## Conclusion

In summary we report the preparation and anti-breast CSC potential of a family of gold(i) complexes comprising of triphenylphosphine and eight different NSAIDs 1–8. The linear geometry of the eight gold(i)-NSAID complexes was unambiguously confirmed by X-ray crystallography. The gold(i) complexes feature a relatively rare Au–O bond, affixing the corresponding NSAID moiety to the gold(i) centre. Three of the complexes 1–3 (containing indomethacin, diflunisal, and naproxen, respectively) were deemed stable in solutions used for cell-based studies. The most active gold(i)-NSAID complex, 1 displayed nanomolar toxicity towards breast CSCs cultured in monolayer systems. The potency of 1 towards breast CSCs was up to three orders of magnitude greater than that of a panel of clinically approved breast cancer drugs (5-fluorouracil, capecitabine, cisplatin, and carboplatin), salinomycin, or any previously reported metal complex. Furthermore, 1 exhibited up to 34-fold and 80-fold greater toxicity towards breast CSCs than non-tumorigenic cells or bulk breast cancer cells, respectively. Strikingly, 1 displayed significantly higher potency for three-dimensional mammosphere cultures than 5-fluorouracil, capecitabine, cisplatin, carboplatin, and salinomycin. Biophysical studies suggest that 1 can rapidly react with thiol-containing biomolecules to form stable gold(i)-biomolecule adducts and simultaneously release indomethacin. Cell-based mechanistic studies indicate that 1 readily enters breast CSCs, localises in the cytoplasm, elevates intracellular ROS levels, inhibits COX-2 activity, and prompts apoptosis. Notably 1 inhibited tumour progression in an immunocompetent breast cancer mouse model, without inducing weight loss over a 16 days period. Remarkably, histological studies showed that 1 reduced cellularity in tumour tissue but not in kidney or liver tissue. Overall, the *in vivo* studies suggest that 1 is an efficacious antiproliferative agent with promising translatable scope. Not only does this study reinforce the therapeutic potential of gold(i) complexes but it also provides the basis for the development of other gold coordination complexes as breast CSC potent and selective drug candidates.

## Data availability

All related data have been provided in the ESI.†

## Author contributions

Conceptualisation, K. S., A. J., and S. G. A.; methodology, A. J., C. O., J. H. K., J. N. S., R. T. M., G. P., K. S., S. G. A., and K. S.; formal analysis, A. J., C. O., J. H. K., J. N. S., R. T. M., G. P., K. S., S. G. A., and K. S.; investigation, A. J., C. O., J. H. K., J. N. S., R. T. M., G. P., K. S., S. G. A., and K. S.; writing—original draft preparation, K. S., A. J., and S. G. A.; writing—review and editing, A. J., C. O., J. H. K., J. N. S., R. T. M., G. P., K. S., S. G. A., and K. S.; supervision, K. S. and S. G. A.; project administration, K. S. and S. G. A.; funding acquisition, K. S. and S. G. A.

## Conflicts of interest

There are no conflicts to declare.

## Supplementary Material

SC-014-D2SC04707A-s001

SC-014-D2SC04707A-s002
