# Supplementary material for: The anti-breast cancer stem cell properties of gold(i)-non-steroidal anti-inflammatory drug complexes
Source: Chem Sci. 2022 Dec 12;14(3):557–65. doi: 10.1039/d2sc04707a (PMC9847679; doi:10.1039/d2sc04707a)
Supplement: SC-014-D2SC04707A-s001 [file SC-014-D2SC04707A-s001.pdf]

Electronic Supplementary Material (ESI) for Chemical Science.
This journal is © The Royal Society of Chemistry 2022

Supporting Information for

The Anti-Breast Cancer Stem Cell Properties of Gold(I)-Non-Steroidal Anti-Inflammatory Drug Complexes

Alice Johnson,*[a,b] Chibuzor Olelewe,[c] Jong Hyun Kim,[c] Joshua Northcote-Smith,[a] R. Tyler Mertens,[c] Ginevra Passeri,[a] Kuldip Singh,[a] Samuel G. Awuah,*[c,d] and Kogularamanan Suntharalingam*[a]

[a] School of Chemistry, University of Leicester, Leicester, United Kingdom
[b] Biomolecular Sciences Research Centre, Sheffield Hallam University, Sheffield, United Kingdom
[c] Department of Chemistry, University of Kentucky, Lexington, Kentucky, United States
[d] Department of Pharmaceutical Sciences, University of Kentucky, Lexington, Kentucky, United States

* To whom correspondence should be addressed:
Email: k.suntharalingam@leicester.ac.uk; awuah@uky.edu; alice.johnson@shu.ac.uk

Table of Content

Experimental Details
Figure S1. ^{1}H NMR spectrum of **1** in CD_2Cl_2.
Figure S2. $^{31}P\{^{1}H\}$ NMR spectrum of **1** in CD_2Cl_2.
Figure S3. ^{1}H NMR spectrum of **2** in CD_2Cl_2.
Figure S4. $^{31}P\{^{1}H\}$ NMR spectrum of **2** in CD_2Cl_2.
Figure S5. $^{19}F\{^{1}H\}$ NMR spectrum of **2** in $CDCl_3$.
Figure S6. ^{1}H NMR spectrum of **3** in CD_2Cl_2.
Figure S7. $^{31}P\{^{1}H\}$ NMR spectrum of **3** in CD_2Cl_2.
Figure S8. ^{1}H NMR spectrum of **4** in CD_2Cl_2.
Figure S9. $^{31}P\{^{1}H\}$ NMR spectrum of **4** in CD_2Cl_2.
Figure S10. ^{1}H NMR spectrum of **5** in CD_2Cl_2.
Figure S11. $^{31}P\{^{1}H\}$ NMR spectrum of **5** in CD_2Cl_2.
Figure S12. ^{1}H NMR spectrum of **6** in CD_2Cl_2.
Figure S13. $^{31}P\{^{1}H\}$ NMR spectrum of **6** in CD_2Cl_2.
Figure S14. ^{1}H NMR spectrum of **7** in CD_2Cl_2.
Figure S15. $^{31}P\{^{1}H\}$ NMR spectrum of **7** in CD_2Cl_2.
Figure S16. ^{1}H NMR spectrum of **8** in CD_2Cl_2.
Figure S17. $^{31}P\{^{1}H\}$ NMR spectrum of **8** in CD_2Cl_2.
Figure S18. ATR-FTIR spectra of **1-8** (A-H) in the solid form.
Table S1. Crystallographic data for complexes **1-4**.

Table S2. Crystallographic data for complexes **5-8**.

Table S3. Selected bond lengths (Å) and angles (°) for complexes **1-8**.

Figure S19. $^{31}P\{^1H\}$ NMR spectrum of free triphenylphosphine in CD_2Cl_2.

Table S4. Experimentally determined LogP values for Au(I)-NSAID complexes **1-8**.

Figure S20. UV-Vis spectra of **1-8** (A-H) (all 50 μM) in DMSO over the course of 24 h (recorded at 1 h intervals) at 37 °C.

Figure S21. UV-Vis spectra of **1-5** (A-E) (all 50 μM) in PBS:DMSO (1:1) over the course of 24 h (recorded at 1 h intervals) at 37 °C.

Figure S22. UV-Vis spectra of **1-4** (A-D) (all 50 μM) in MEGM:DMSO (1:1) over the course of 24 h (recorded at 1 h intervals) at 37 °C.

Figure S23. $^{31}P\{^1H\}$ NMR spectra of **1** (10 mM) in DMSO-d_6 over the course of 72 h.

Figure S24. $^{31}P\{^1H\}$ NMR spectra of **2** (10 mM) in DMSO-d_6 over the course of 72 h.

Figure S25. $^{31}P\{^1H\}$ NMR spectra of **3** (10 mM) in DMSO-d_6 over the course of 72 h.

Figure S26. ^1H NMR spectra of **1** (10 mM) in DMSO-d_6 over the course of 72 h.

Figure S27. ^1H NMR spectra of **2** (10 mM) in DMSO-d_6 over the course of 72 h.

Figure S28. ^1H NMR spectra of **3** (10 mM) in DMSO-d_6 over the course of 72 h.

Figure S29. $^{31}P\{^1H\}$ NMR spectra of **4** (10 mM) in DMSO-d_6 over the course of 72 h. The $^{31}P\{^1H\}$ NMR spectrum of O=PPh_3 (10 mM) in DMSO-d_6 is also provided.

Figure S30. ^1H NMR spectra of **4** (10 mM) in DMSO-d_6 over the course of 72 h.

Figure S31. Representative dose-response curves for the treatment of MDA-MB-231, MDA-MB-468, and 4T1 cells with **1** after 72 h incubation.

Figure S32. Representative dose-response curves for the treatment of MDA-MB-231, MDA-MB-468, and 4T1 cells with **2** after 72 h incubation.

Figure S33. Representative dose-response curves for the treatment of MDA-MB-231, MDA-MB-468, and 4T1 cells with **3** after 72 h incubation.

Figure S34. Representative dose-response curves for the treatment of HMLER and HMLER-shEcad cells with **1** after 72 h incubation.

Figure S35. Representative dose-response curves for the treatment of HMLER and HMLER-shEcad cells with **2** after 72 h incubation.

Figure S36. Representative dose-response curves for the treatment of HMLER and HMLER-shEcad cells with **3** after 72 h incubation.

Table S5. IC_{50} values of the gold(I)-NSAID complexes, **1-3** against MDA-MB-231, MDA-MB-468, and 4T1 cells. [a]Determined after 72 h incubation (mean of three independent experiments ± SD).

Figure S37. Representative dose-response curves for the treatment of HMLER and HMLER-shEcad cells with 5-fluorouracil after 72 h incubation.

Figure S38. Representative dose-response curves for the treatment of HMLER and HMLER-shEcad cells with capecitabine after 72 h incubation.

Figure S39. Representative dose-response curves for the treatment of HEK 293 cells with **1** after 72 h incubation.

Figure S40. Representative dose-response curves for the treatment of HMLER and HMLER-shEcad cells with indomethacin after 72 h incubation.

Figure S41. Representative dose-response curves for the treatment of HMLER and HMLER-shEcad cells with chloro(triphenylphosphine)gold(I) after 72 h incubation.

Figure S42. Representative dose-response curves for the treatment of HMLER and HMLER-shEcad cells with a 1:1 mixture of indomethacin and chloro(triphenylphosphine)gold(I) after 72 h incubation.

Figure S43. Representative dose-response curves for the treatment of HMLER and HMLER-shEcad cells with auranofin after 72 h incubation.

Table S6. IC_{50} values of indomethacin, chloro(triphenylphosphine)gold(I), a 1:1 mixture of indomethacin and chloro(triphenylphosphine)gold(I), and auranofin against HMLER and HMLER-shEcad cells. [a]Determined after 72 h incubation (mean of three independent experiments ± SD).
Figure S44. Representative bright-field images (x 10) of the mammospheres in the absence and presence of 5-fluorouracil, capecitabine, cisplatin, carboplatin, and salinomycin at their respective IC_{20} values after 5 day incubation.
Figure S45. Representative dose-response curves for the treatment of HMLER-shEcad mammospheres with **1**-**3** after 5 days incubation.
Figure S46. Representative dose-response curves for the treatment of HMLER-shEcad mammospheres with 5-fluorouracil and capecitabine after 5 days incubation.
Figure S47. Gold content (ng of Au/ 10^6 cells) in HMLER-shEcad cells after treatment with **1**-**3** (0.25 µM for 24 h).
Figure S48. Gold content (ng of Au/ 10^6 cells) in various cellular components upon treatment of HMLER-shEcad cells with **1** (0.25 µM for 24 h).
Figure S49. ^1H NMR spectra for complex **1** (10 mM) in DMSO-d_6, in the absence and presence of glutathione (GSH, 10 mM) over the course of 72 h at 37 °C. The ^1H NMR spectra of indomethacin and $[Au^I(GSH)(PPh_3)]$ (both 10 mM) in DMSO-d_6 are also provided.
Figure S50. $^{31}P\{^1H\}$ NMR spectra for complex **1** (10 mM) in DMSO-d_6, in the absence and presence of *N*-acetylcysteine (NAC, 10 mM) over the course of 72 h at 37 °C. The $^{31}P\{^1H\}$ NMR spectrum of $[Au^I(NAC)(PPh_3)]$ (10 mM) in DMSO-d_6 is also provided.
Figure S51. $^{31}P\{^1H\}$ NMR spectra for complex **1** (10 mM) in DMSO-d_6, in the absence and presence of glutathione (GSH, 10 mM) over the course of 72 h at 37 °C. The $^{31}P\{^1H\}$ NMR spectrum of $[Au^I(GSH)(PPh_3)]$ (10 mM) in DMSO-d_6 is also provided.
Scheme S1. Representative scheme for the reaction of **1** with glutathione.
Figure S52. Representative histograms displaying the green fluorescence emitted by DCFH-DA-stained HMLER-shEcad cells (red) and HMLER-shEcad cells treated with **1** (0.4 µM) (blue) for (A) 1 h, (B) 3 h, (C) 6 h, (D) 16 h, and (E) 24 h.
Figure S53. Summarised ROS data. Normalised ROS activity in untreated HMLER-shEcad cells (control) and HMLER-shEcad cells treated with **1** (0.4 µM for 1, 3, 6, 16, and 24 h).
Figure S54. Representative dose-response curves for the inhibition of COX-2 activity by **1**, indomethacin, or chloro(triphenylphosphine)gold(I).
Table S7. IC_{50} values of **1**, indomethacin, and chloro(triphenylphosphine)gold(I) associated to their ability to inhibit COX-2 activity.
References

Experimental Details

Instrumentation. ^{1}H, $^{13}C\{^{1}H\}$, $^{31}P\{^{1}H\}$ and $^{19}F\{^{1}H\}$ NMR were recorded at room temperature on a Bruker Avance 400 spectrometer (^{1}H 400.0 MHz, ^{13}C 100.6 MHz, ^{31}P 162.0 MHz, ^{19}F 376.5 MHz) with chemical shifts (δ, ppm) reported relative to the solvent peaks of the deuterated solvent. All *J* values are given in Hz. ICP-MS were measured using a Thermo Scientific ICAP-Qc quadrupole ICP mass spectrometer. Elemental Analysis was performed commercially at the University of Cambridge.

Starting Materials. Silver(I) nitrate, chloro(triphenylphosphine)gold(I), and the sodium salts of the NSAIDs (naproxen, diclofenac, and salicylic acid) were purchased from Sigma-Aldrich and used without further purification. Solvents were purchased from Fisher and used without further purification. Sodium salts of indomethacin, diflunisal, tolfenamic acid, mefenamic acid, and ibuprofen were prepared as follows: to a solution of indomethacin, diflunisal, tolfenamic acid, mefenamic acid, or ibuprofen (1 mmol) in MeOH (10 mL) was added NaOH (1M MeOH, 1 mmol) and the solution stirred for 1 h. The solution was concentrated to dryness under vacuum and the solid washed with diethyl ether and dried under vacuum to give the sodium salts of the respective NSAIDs. Sodium salt of indomethacin, yellow solid, 347.9 mg, 92%; Sodium salt of diflunisal, white solid, 258.2 mg, 95%; Sodium salt of tolfenamic acid, white solid, 231.4 mg, 82%; Sodium salt of mefenamic acid, white solid, 230.4 mg, 88%; Sodium salt of ibuprofen, white solid, 216.2 mg, 95%.

General Synthesis of the Gold(I)-NSAID Triphenylphosphine Complexes, 1-8. To a solution of the corresponding NSAID sodium salt (0.1 mmol) in H_2O (10 mL) was added $AgNO_3$ (17.00 mg, 0.1 mmol) and the mixture stirred for 1 h in the dark. The corresponding insoluble Ag(I)-NSAID complex was collected by filtration and washed with H_2O (10 mL), diethyl ether (10 mL) and acetone (10 mL), and dried under vacuum. To a solution of $AuCl(PPh_3)$ (49.4 mg, 0.1 mmol) in dichloromethane (10 mL) was added the corresponding Ag(I)-NSAID complex (0.1 mmol) and the mixture stirred for 2 h in the dark. The solution was filtered through celite and the filtrate concentrated to minimum volume under vacuum. Diethyl ether (10 mL) was added to precipitate a white or yellow solid which was collected and dried under vacuum to give the corresponding Au(I)-NSAID complex **1-8**.

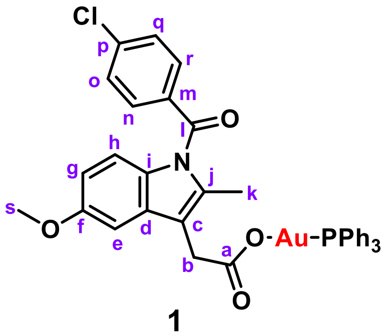

1

Au(I)-NSAID complex 1: yellow solid, 54.7 mg, 67%; ^{1}H NMR (400 MHz, CD_2Cl_2) δ 7.83 – 7.24 (m, 19H, PPh_3 + $CH_{n+o+q+r}$), 7.07 (s, 1H, CH_e), 6.97 (d, *J* = 8.6 Hz, 1H, CH_h), 6.64 (d, *J* = 8.6 Hz, 1H, CH_g), 3.81 (s, 3H, CH_s), 3.63 (s, 2H, CH_b), 2.33 (s, 3H, CH_k); ^{31}P NMR (162 MHz, CD_2Cl_2) δ 27.37 (s, PPh_3); ATR-FTIR (solid, cm^{-1}): 3056, 2928, 2832, 1674, 1626, 1477, 1433, 1355, 1315, 1223, 1179, 1144, 1100, 1087, 1065, 1035, 1013, 995, 921, 833, 750, 710, 693, 548, 505, 483, 434; Anal. Calcd. $C_{37}H_{30}AuClNO_4P$: C 54.46, H 3.71, N 1.72, Found: C 54.83, H 3.73, N 1.70.

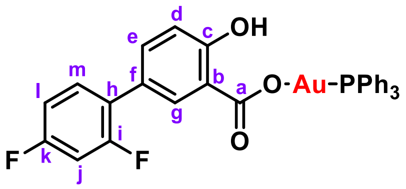

2

Au(I)-NSAID complex 2: white solid, 41.1 mg, 58%; ^{1}H NMR (400 MHz, CD_2Cl_2) δ 12.40 (s, 1H, OH), 8.08 (s, 1H, CH_g), 7.63 – 7.51 (m, 16H, PPh_3 + CH_e), 7.46 – 7.40 (m, 1H, CH_m), 6.99 – 6.89 (m, 3H, CH_{d+j+l}); ^{31}P NMR (162 MHz, CD_2Cl_2) δ 27.55 (s, PPh_3); ^{19}F NMR (376 MHz, CD_2Cl_2) δ -113.22 (d, $^4J_{FF}$ = 6.5 Hz, CF), -114.42 (d, $^4J_{FF}$ = 6.5 Hz, CF); ATR-FTIR (solid, cm^{-1}): 3072, 1632, 1562, 1431, 1365, 1299, 1251, 1221, 1142, 1102, 997, 967, 836, 809, 752, 713, 691, 669, 590, 547, 529, 512, 499, 477, 464, 433; Anal. Calcd. $C_{31}H_{22}AuF_2O_3P{\cdot}H_2O$: C 51.25, H 3.33, N 0.00; Found: C 51.09, H 2.98, N 0.00.

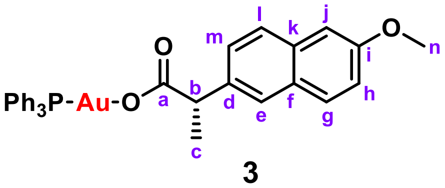

3

Au(I)-NSAID complex 3: white solid, 44.1 mg, 64%; ^{1}H NMR (400 MHz, CD_2Cl_2) δ 7.72 – 7.67 (m, 3H, CH_{e+g+l}), 7.56 – 7.44 (m, 16H, PPh_3 + CH_m), 7.15 – 7.06 (m, 2H, CH_{h+j}), 3.90 (s, 3H, CH_n), 3.81 (q, $^3J_{HH}$ = 7.2 Hz, 1H, CH_b), 1.53 (s, 3H, CH_c); ^{31}P NMR (162 MHz, CD_2Cl_2) δ 27.40 (s, PPh_3); ATR-FTIR (solid, cm^{-1}): 3056, 2960, 2928, 1633, 1603, 1482, 1430, 1327, 1262, 1236, 1205, 1158, 1102, 1032, 998, 924, 885, 851, 807, 760, 743, 712, 691, 548, 505, 474, 440, 405; Anal. Calcd. $C_{32}H_{28}AuO_3P$: C 55.82, H 4.10, N 0.00; Found: C 55.93, H 4.04, N 0.00.

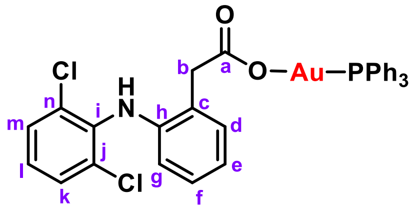

4

Au(I)-NSAID complex 4: white solid, 50.8 mg, 67%; ^{1}H NMR (400 MHz, CD_2Cl_2) δ 8.15 (s, 1H, NH), 7.63 – 7.45 (m, 15H, PPh_3), 7.35 (d, J = 8.1 Hz, 2H, CH_{k+m}), 7.23 (d, J = 6.6 Hz, 1H, CH_g), 7.09 – 7.02 (m, 1H, CH_f), 6.99 (t, J = 8.1 Hz, 1H, CH_l), 6.88 (t, J = 7.1 Hz, 1H, CH_e), 6.46 (d, J = 8.0 Hz, 1H, CH_d), 3.73 (s, 2H, CH_b); ^{31}P NMR (162 MHz, CD_2Cl_2) δ 27.52 (s, PPh_3); ATR-FTIR (solid, cm^{-1}): 3235, 3054, 1648, 1577, 1510, 1479, 1452, 1435, 1306, 1150, 1101, 999, 861, 767, 745, 714, 692, 660, 549, 500, 478, 447; Anal. Calcd. $C_{32}H_{25}AuCl_2NO_2P$: C 50.95, H 3.34, N 1.86; Found: C 50.77, H 3.20, N 2.02.

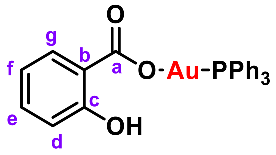

5

Au(I)-NSAID complex 5: white solid, 40.5 mg, 68%; ^{1}H NMR (400 MHz, CD_2Cl_2) δ 12.28 (s, 1H, OH), 7.90 (dd, J = 7.8, 1.7 Hz, 1H, CH_g), 7.71 – 7.43 (m, 15H, PPh_3), 7.43 – 7.30 (m, 1H, CH_e), 6.88 (dd, J = 8.3, 0.8 Hz, 1H, CH_d), 6.85 – 6.77 (m, 1H, CH_f); ^{31}P NMR (162 MHz, CD_2Cl_2) δ 27.55 (s, PPh_3); ATR-FTIR (solid, cm^{-1}): 3046, 1630, 1584, 1564, 1478, 1430, 1375, 1351, 1306, 1244, 1223, 1154, 1100, 1027, 998, 863, 809, 768, 744, 690, 666, 547, 502, 448, 415; Anal. Calcd. $C_{25}H_{20}AuO_3P$: C 50.35, H 3.38, N 0.00; Found: C 50.17, H 3.32, N 0.00.

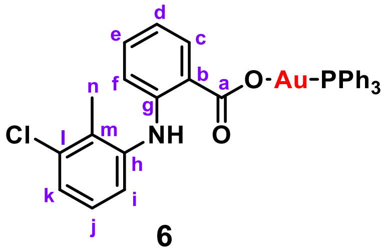

6

Au(I)-NSAID complex 6: white solid, 47.2 mg, 66%; ^{1}H NMR (400 MHz, CD_2Cl_2) δ 10.02 (s, 1H, NH), 8.05 (dd, J = 7.9, 1.6 Hz, 1H, CH_c), 7.67 – 7.44 (m, 15H, PPh_3), 7.34 – 7.19 (m, 2H, CH_{e+k}), 7.19 – 7.04 (m, 2H, CH_{i+j}), 6.93 – 6.88 (m, 1H, CH_f), 6.72 (ddd, J = 8.2, 7.1, 1.1 Hz, 1H, CH_d), 2.35 (s, 3H, CH_n); ^{31}P NMR (162 MHz, CD_2Cl_2) δ 27.65 (s, PPh_3); ATR-FTIR (solid, cm^{-1}): 3202, 1620, 1579, 1562, 1500, 1430, 1347, 1263, 1150, 1102, 996, 786, 747, 690, 672, 611, 549, 497, 444; Anal. Calcd. $C_{32}H_{26}AuClNO_2P$: C 53.39, H 3.64, N 1.95. Found: C 53.36, H 3.65, N 2.01.

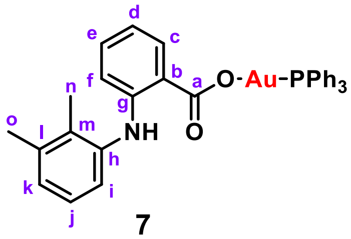

7

Au(I)-NSAID complex 7: white solid, 55.3 mg, 79%; ^{1}H NMR (400 MHz, CD_2Cl_2) δ 9.82 (s, 1H, NH), 8.03 (dd, J = 7.9, 1.6 Hz, 1H, CH_e), 7.67 – 7.45 (m, 15H, PPh_3), 7.23 – 7.11 (m, 2H, CH_{c+i}), 7.06 (t, $^{3}J_{HH}$ = 7.7 Hz, 1H, CH_j), 6.95 (d, $^{3}J_{HH}$ = 7.4 Hz, 1H, CH_k), 6.76 (d, $^{3}J_{HH}$ = 7.8 Hz, 1H, CH_f), 6.70 – 6.59 (m, 1H, CH_d), 2.31 (s, 3H, CH_o), 2.19 (s, 3H, CH_n); ^{31}P NMR (162 MHz, CD_2Cl_2) δ 27.67 (s, PPh_3); ATR-FTIR (solid, cm^{-1}): 3251, 3069, 1621, 1578, 1496, 1432, 1346, 1264, 1157, 1101, 998, 852, 783, 745, 689, 547, 500, 448, 431; Anal. Calcd. $C_{33}H_{29}AuNO_2P$: C 56.66, H 4.18, N 2.00; Found: C 56.45, H 4.15, N 2.15.

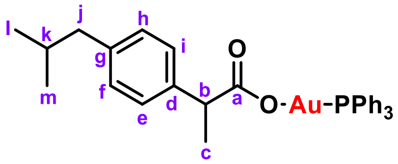

8

Au(I)-NSAID complex 8: white solid, 23.2 mg, 35%; ^{1}H NMR (400 MHz, CD_2Cl_2) δ 7.72 – 7.35 (m, 15H, PPh_3), 7.26 (d, J = 7.9 Hz, 2H, CH_{f+h}), 7.07 (d, J = 7.9 Hz, 2H, CH_{e+i}), 3.64 (q, J = 7.2 Hz, 1H, CH_b), 2.44 (d, J = 7.2 Hz, 2H, CH_j), 1.91 – 1.78 (m, 1H, CH_k), 1.44 (d, J = 7.2 Hz, 3H, CH_c), 0.90 (d, J = 6.6 Hz, 6H, CH_{l+m}); ^{31}P NMR (162 MHz, CD_2Cl_2) δ 27.42 (s, PPh_3); ATR-FTIR (solid, cm^{-1}): 3051, 2955, 2859, 1632, 1479, 1432, 1372, 1312, 1233, 1181, 1098, 996, 879, 847, 745, 712, 689, 545, 503, 438; Anal. Calcd. $C_{31}H_{32}AuO_2P{\cdot}H_2O$: C 54.55, H 5.02, N 0.00; Found: C 54.61, H 4.69, N 0.00.

X-ray Single Crystal Diffraction Analysis. Single crystals of **1-8** were obtained by layer-diffusion of hexane into a DCM solution of the gold(I)-NSAID complex. Crystals suitable for X-ray diffraction analysis were selected and mounted on a Bruker Apex 2000 CCD area detector diffractometer using standard procedures. Data was collected using graphite-monochromated Mo-Kα radiation (λ = 0.71073) at 150(2) K. Crystal structures were solved and refined using the Bruker SHELXTL software.[1] All hydrogen atoms were located by geometrical calculations, and all non-hydrogen atoms were refined anisotropically. The structures have been deposited with the Cambridge Crystallographic Data Centre (CCDC 2175736-2175743). This information can be obtained free of charge from www.ccdc.cam.ac.uk/data_request/cif.

Measurement of water-octanol partition coefficient (LogP). The LogP values for **1-8** were determined using the shake-flask method and UV-vis spectroscopy. The 1-octanol used in this experiment was pre-saturated with water. An aqueous solution of **1-8** (500 μL, 100 μM) was incubated with 1-octanol (500 μL) in a 1.5 mL tube. The tube was shaken at room temperature for 24 h. The two phases were separated by centrifugation and the **1-8** content in each phase was determined by UV-vis spectroscopy.

Cell Lines and Cell Culture Conditions. The human mammary epithelial HMLER and HMLER-shEcad cell lines were kindly donated by Prof. R. A. Weinberg (Whitehead Institute, MIT). The human epithelial breast MCF10A, breast adenocarcinoma MDA-MB-231 and MDA-MB-468, murine epithelial breast 4T1, and embryonic kidney HEK 293 cell lines were acquired from American Type Culture Collection (ATCC, Manassas, VA, USA). HMLER, HMLER-shEcad, and MCF10A cells were maintained in Mammary Epithelial Cell Growth Medium (MEGM) with supplements and growth factors (BPE, hydrocortisone, hEGF, insulin, and gentamicin/amphotericin-B). MDA-MB-231, MDA-MB-468, 4T1, and HEK 293 cells were maintained in Dulbecco's Modified Eagle's Medium (DMEM) supplemented with fetal bovine serum (10 %) and penicillin/streptomycin (1 %). The cells were grown at 310 K in a humidified atmosphere containing 5% CO_2.

Monolayer Cytotoxicity Studies. Exponentially growing cells were seeded at a density of approximately 5×10^3 cells per well in 96-well flat-bottomed microplates and allowed to attach for 24 h prior to addition of compounds. Various concentrations of the test compounds (0.0004-100 μM) were added and incubated for 72 h at 37 °C (total volume 200 μl). Stock solutions of the compounds were prepared as 10 mM DMSO solutions and diluted using cell

media. The final concentration of DMSO in each well was ≤1 %. After 72 h, 20 μl of MTT (4 mg mL^{-1} in PBS) was added to each well and the plates incubated for an additional 4 h at 37 °C. The media/MTT mixture was eliminated and DMSO (100 μl per well) was added to dissolve the formazan precipitates. The optical density was measured at 550 nm using a 96-well multiscanner autoreader. Absorbance values were normalised to (DMSO-containing) control wells and plotted as concentration of test compound versus % cell viability. IC_{50} values were interpolated from the resulting dose dependent curves. The reported IC_{50} values are the average of three independent experiments (n = 18).

Tumorsphere Formation and Viability Assay. HMLER-shEcad cells (5×10^3) were plated in ultralow-attachment 96-well plates (Corning) and incubated in MEGM supplemented with B27 (Invitrogen), 20 ng mL^{-1} EGF and 4 μg mL^{-1} heparin (Sigma) for 5 days. Studies were also conducted in the presence of **1-3**, 5-fluorouracil, capecitabine, cisplatin, carboplatin, and salinomycin (0-100 μM). Mammospheres treated with **1-3**, 5-fluorouracil, capecitabine, cisplatin, carboplatin, and salinomycin (at their respective IC_{20} values, 5 days) were counted and imaged using an inverted microscope. The viability of the mammospheres was determined by addition of a resazurin-based reagent, TOX8 (Sigma). After incubation for 16 h, the fluorescence of the solutions was read at 590 nm (λ_{ex} = 560 nm). Viable mammospheres reduce the amount of the oxidised TOX8 form (blue) and concurrently increase the amount of the fluorescent TOX8 intermediate (red), indicating the degree of mammosphere cytotoxicity caused by the test compound. Fluorescence values were normalised to DMSO-containing controls and plotted as concentration of test compound versus % mammospheres viability. IC_{50} values were interpolated from the resulting dose dependent curves. The reported IC_{50} values are the average of three independent experiments, each consisting of two replicates per concentration level (overall n = 6).

Cellular Uptake. To measure the cellular uptake of **1-3** about 1 million HMLER-shEcad cells were treated with **1-3** (0.25 μM) at 37 °C for 24 h. After incubation, the media was removed, the cells were washed with PBS (2 mL × 3) and harvested. The number of cells was counted at this stage, using a haemocytometer. This mitigates any cell death induced by **1-3** at the administered concentration and experimental cell loss. The cellular pellets were dissolved in 65% HNO_3 (250 μL) overnight. A cellular pellet of HMLER-shEcad cells treated with **1** was also used to determine the gold content in the nuclear, cytoplasmic and membrane fractions. The Thermo Scientific NE-PER Nuclear and Cytoplasmic Extraction Kit was used to extract and separate the nuclear, cytoplasmic and membrane fractions. The fractions were dissolved in 65% HNO_3 (250 μL final volume) overnight. All samples were diluted 17-fold with water and analysed using inductively coupled plasma mass spectrometry (ICP-MS, Thermo Scientific ICAP-Qc quadrupole ICP mass spectrometer). Gold levels are expressed as mass of Au (ng) per million cells. Results are presented as the mean of four determinations for each data point.

Intracellular ROS Assay. Untreated and **1**-treated (0.4 μM for 1-24 h) HMLER-shEcad cells (5×10^5 cells/well) grown in six-well plates were incubated with 2′,7′-dichlorodihydro-fluorescein diacetate (DCFH-DA) (20 μM) for 30 min. The cells were then washed with PBS (1 mL), harvested by trypsinisation, and analysed using a FACSCanto II flow cytometer (BD Biosciences) (10,000 events per sample were acquired) at the University of Leicester FACS Facility. The FL1 channel was used to assess intracellular ROS levels. Cell populations were analysed using the FlowJo software (Tree Star).

Annexin V-Propidium Iodide Assay. HMLER-shEcad cells were incubated with and without **1** (IC_{50} value × 2 for 48 h) and cisplatin (25 µM for 48 h) at 37 °C. Cells were harvested from adherent cultures by trypsinisation. The FITC Annexin V/Dead Cell Apoptosis Kit was used. The manufacture's (Thermo Fisher Scientific) protocol was followed to carry out this experiment. Briefly, untreated and treated cells (1×10^6) were suspended in 1× Annexin binding buffer (100 µL) (10 mM HEPES, 140 mM NaCl, 2.5 mM $CaCl_2$, pH 7.4), then 5 µL FITC Annexin V and 1 µL PI (100 µg/ mL) were added to each sample and incubated at room temperature for 15 min. After which more 1× Annexin binding buffer (400 µL) was added while gently mixing. The cells were analysed using a FACSCanto II flow cytometer (BD Biosciences) (10,000 events per sample were acquired) at the University of Leicester FACS Facility. The FL1 channel was used to assess Annexin V binding and the FL2 channel was used to assess PI uptake. Cell populations were analysed using the FlowJo software (Tree Star).

COX-2 Expression Assay. HMLER-shEcad cells were seeded in 6-well plates (at a density of 5×10^5 cells/ mL) and the cells were allowed to attach overnight. The cells were treated with lipopolysaccharide (LPS) (2.5 µM for 24 h), and then treated with **1** (IC_{50} value) or indomethacin (20 µM) and incubated for a further 48 h. The cells were then harvested by trypsinisation, fixed with 4% paraformaldehyde (at 37 °C for 10 min), permeabilised with ice-cold methanol (for 30 min), and suspended in PBS (200 µL). The Alexa Fluor® 488 nm labelled anti-COX-2 antibody (5 µL) was then added to the cell suspension and incubated in the dark for 1 h. The cells were then washed with PBS (1 mL) and analysed using a FACSCanto II flow cytometer (BD Biosciences) (10,000 events per sample were acquired) at the University of Leicester FACS Facility. The FL1 channel was used to assess COX-2 expression. Cell populations were analysed using the FlowJo software (Tree Star).

COX-2 Inhibition Enzyme Immunoassay (EIA). The COX-2 inhibitory properties of **1**, indomethacin, and chloro(triphenylphosphine)gold(I) was determined using the "COX (ovine) Inhibitor Screening Assay (Cayman Chemicals)." The assay was performed according to the manufacturer's instructions using a 96-well plate set up. The inhibitory activity was determined between 1-250 µM for all of the compounds tested. The absorption was recorded using a microplate reader at 420 nm.

Mice. Female, 5-week-old BALB/c mice were purchased from Charles River Laboratories (Wilmington, MA). All mice were quarantined for one week before use and kept in micro-isolator cages (four mice per cage) in a temperature- and humidity-controlled environment as per the Division of Laboratory Animal Research (DLAR) of University of Kentucky. All mice were maintained in a pathogen-free environment under the care of DLAR of University of Kentucky. Our study was performed in compliance with the NIH guidelines (NIH Publication No. 85-23 Rev. 1985) for the care and use of laboratory animals and all experimental procedures were monitored and approved by the Institutional Animal Care and Use Committee (IACUC) of University of Kentucky (USA).

***In vivo* studies with 1**. Ten female BALB/c mice (5 weeks) were received from Charles River Laboratories (Wilmington, MA). They had an acclimation period of one week before being implanted with 4T1 cells (1 million) subcutaneously on their right flank. Three days post-implantation, the mice were administered with 10 mg/kg of **1** intraperitoneally (IP), 0.1 mL/mouse formulated as 1% DMSO, 10% Kolliphor, and 89% PBS. The control group was injected with a PBS solution containing 1% DMSO and 1% Kolliphor. The injection of **1**, and

tumour-size and body-weight measurements were performed three days a week, and the mice were euthanised after 16 days (n = 5 for **1** and n = 5 for vehicle control).

Tissue biodistribution of 1. Following the treatment of mice with **1** for 16 days, each tissue (heart, lung, liver, spleen, kidney, and tumour) was excised from euthanised mice (n = 3). The tissues were boiled for 5 h at 60 °C with 70% nitric acid (0.5 mL), and then boiled again at 60 °C for 10 min with 35% hydrogen peroxide (0.5 mL). The solutions turned yellow, and were diluted as needed to measure the gold content using a Graphite Furnace Atomic Absorption Spectrometer. Before measuring all samples, the standard solution curves were measured.

Hematoxylin and eosin staining. The mice used in the *in vivo* comparative experiment of **1** and the vehicle control were sacrificed at day 16 post tumour cells (4T1) injection. The tissues (liver, kidney, and tumour) were fixed in freshly prepared paraformaldehyde (4% in PBS) for 24 h and processed for paraffin sectioning. Tissue sections of 5 µm were stained with hematoxylin and eosin and used for histological examination. A total of 3 sections per tissue (spanning the full depth of the tissue) were examined and photographed using a Nikon Eclipse 55i microscope.

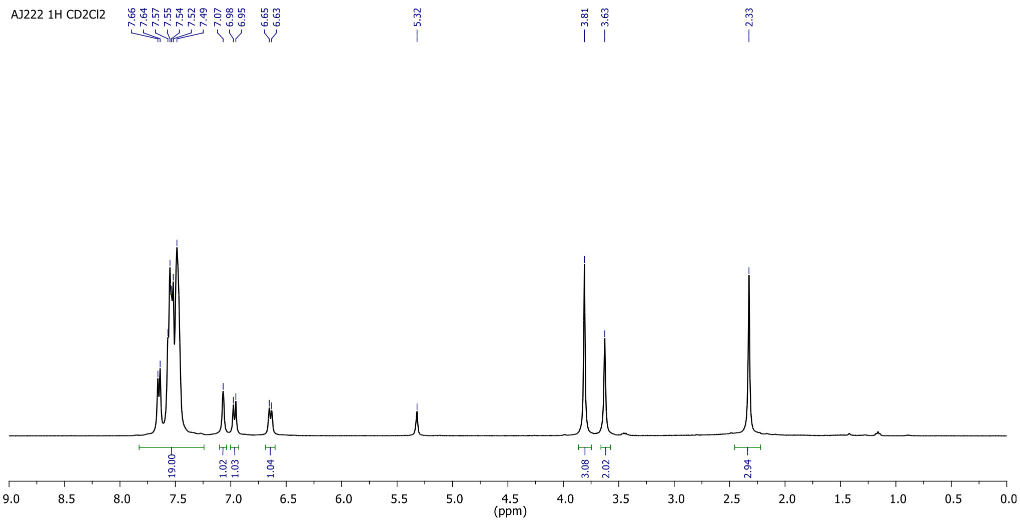

Figure S1. ^{1}H NMR spectrum of **1** in CD_2Cl_2.

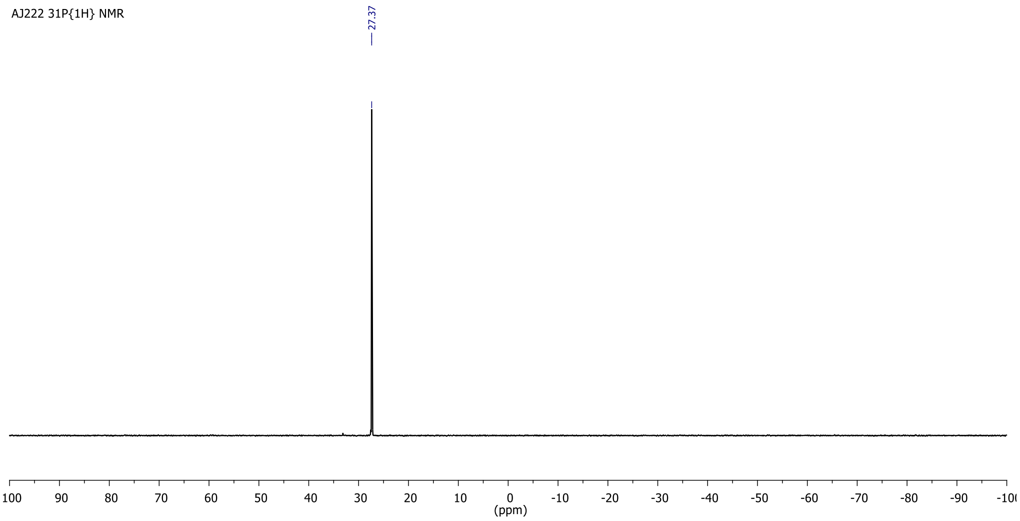

Figure S2. $^{31}P\{^{1}H\}$ NMR spectrum of **1** in CD_2Cl_2.

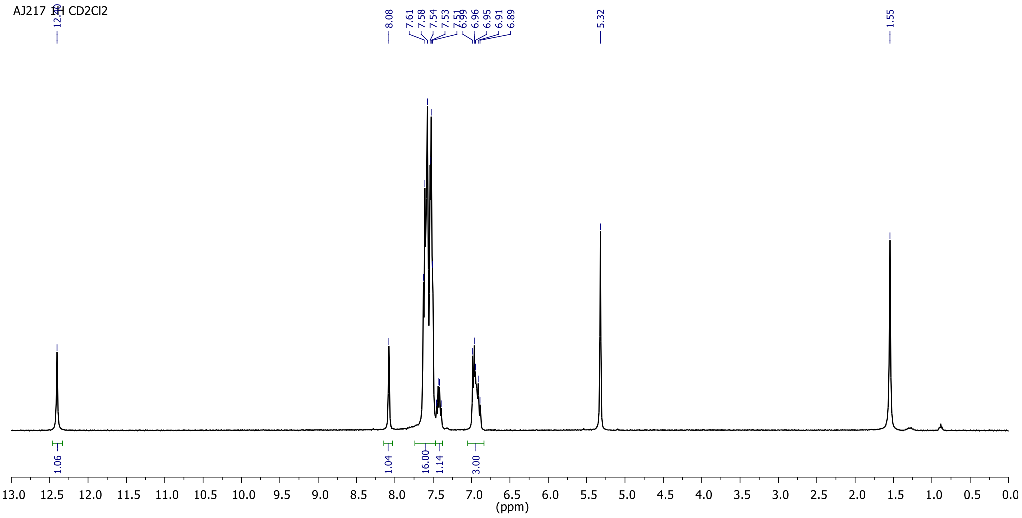

Figure S3. ^{1}H NMR spectrum of **2** in CD_2Cl_2.

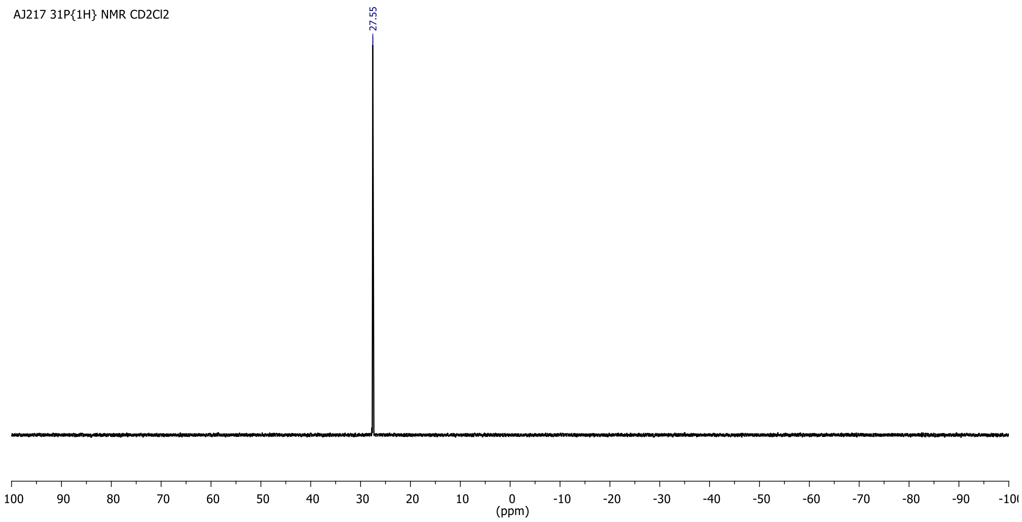

Figure S4. $^{31}P\{^{1}H\}$ NMR spectrum of **2** in CD_2Cl_2.

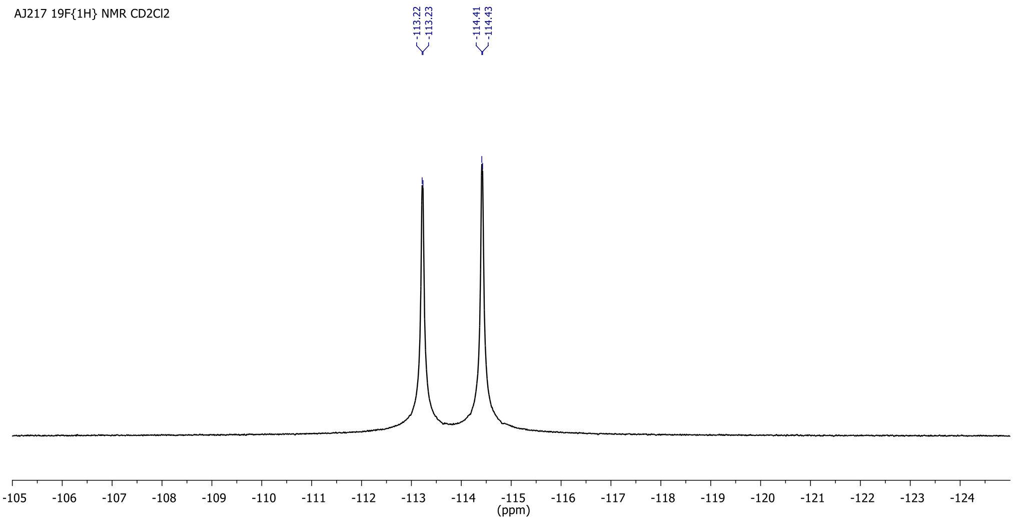

Figure S5. $^{19}F\{^{1}H\}$ NMR spectrum of **2** in $CDCl_3$.

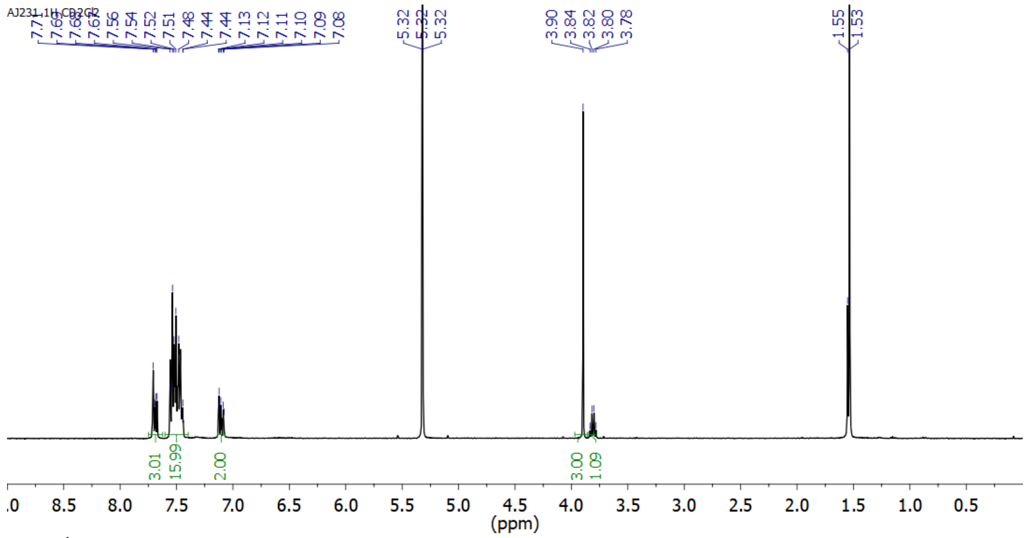

Figure S6. ^{1}H NMR spectrum of **3** in CD_2Cl_2.

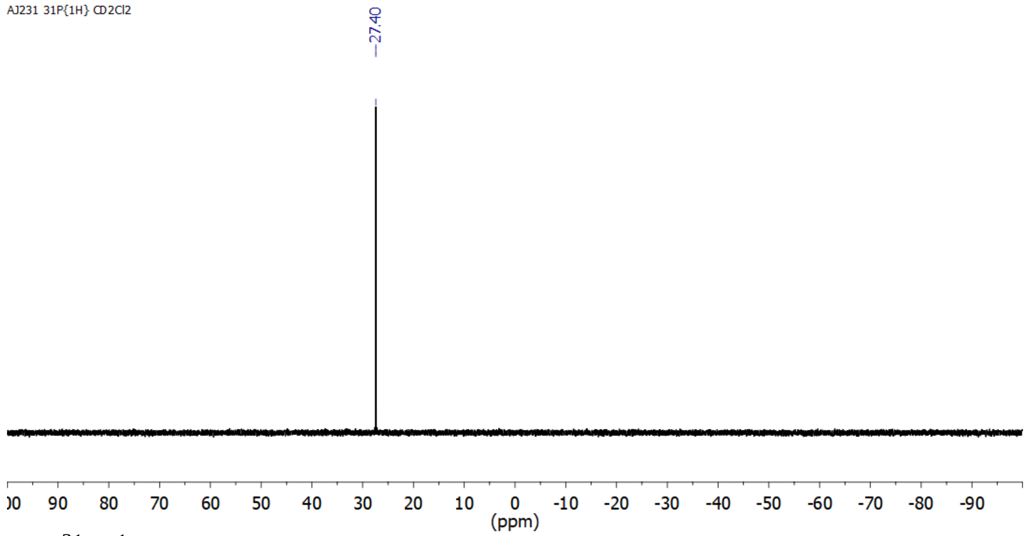

Figure S7. $^{31}P\{^{1}H\}$ NMR spectrum of **3** in CD_2Cl_2.

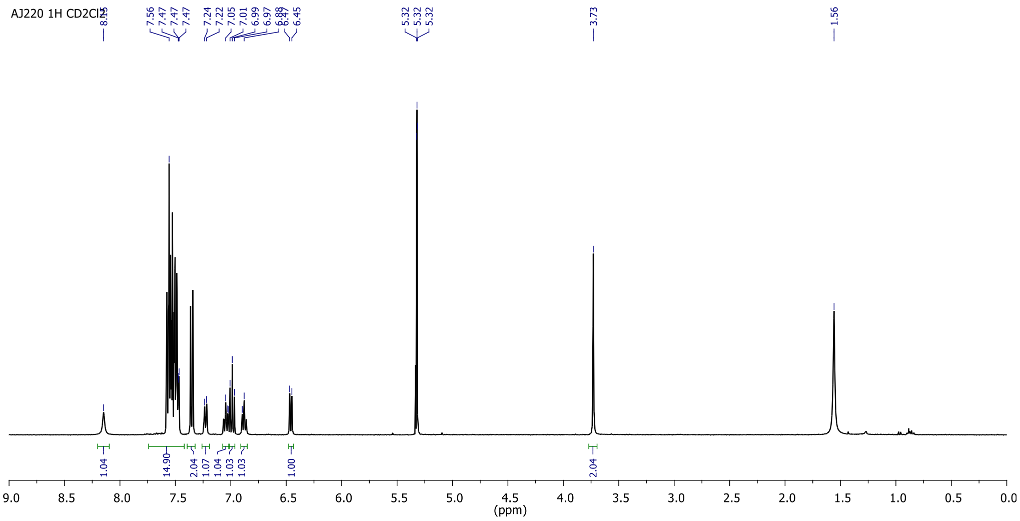

Figure S8. ^{1}H NMR spectrum of **4** in CD_2Cl_2.

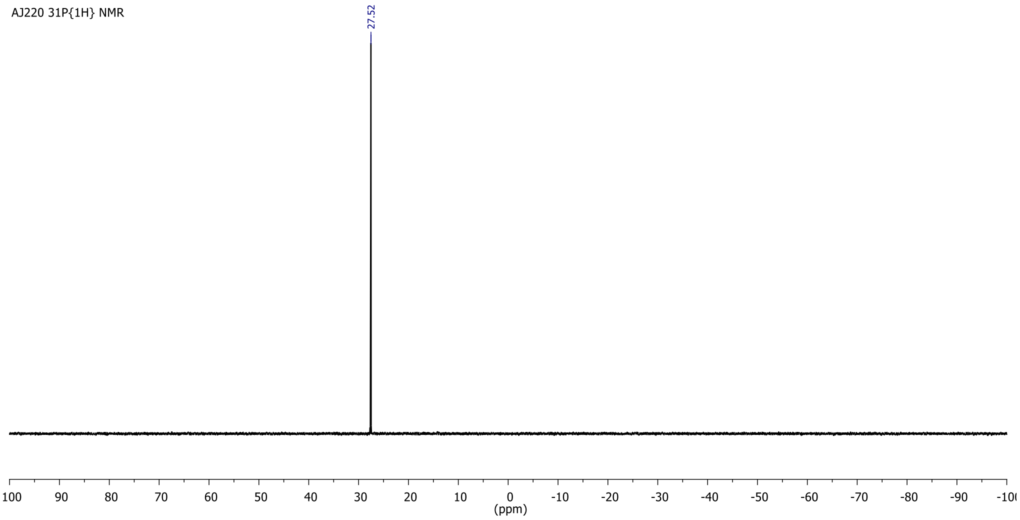

Figure S9. $^{31}P\{^{1}H\}$ NMR spectrum of **4** in CD_2Cl_2.

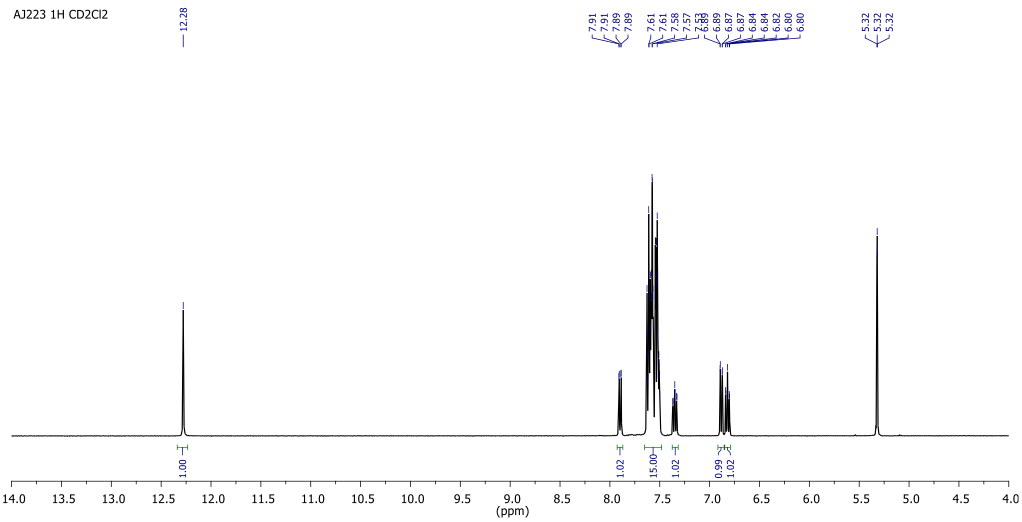

Figure S10. ^{1}H NMR spectrum of **5** in CD_2Cl_2.

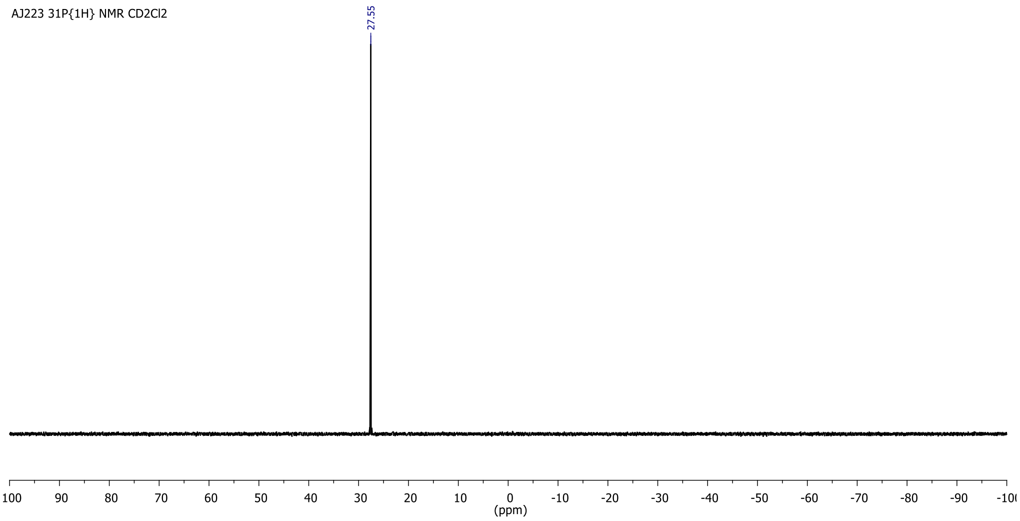

Figure S11. $^{31}P\{^{1}H\}$ NMR spectrum of **5** in CD_2Cl_2.

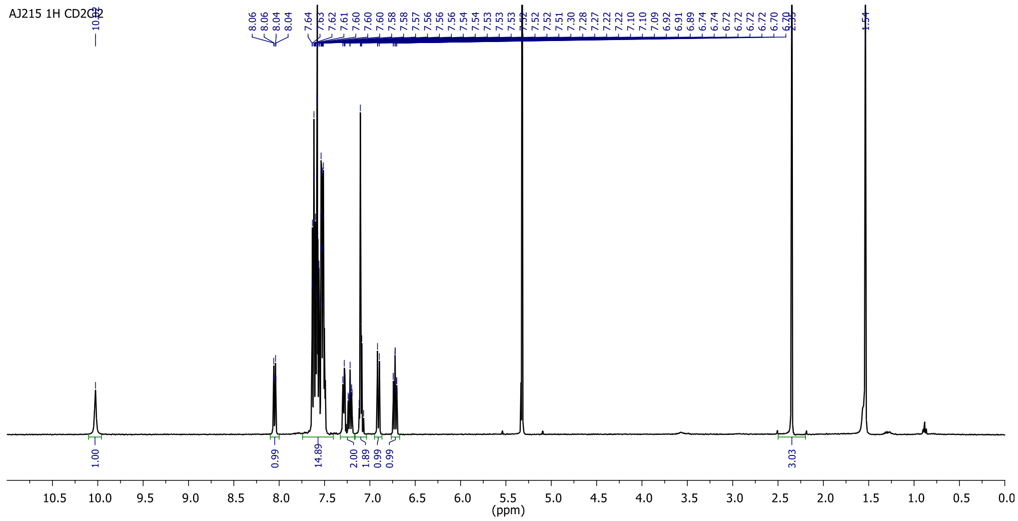

Figure S12. ^{1}H NMR spectrum of **6** in CD_2Cl_2.

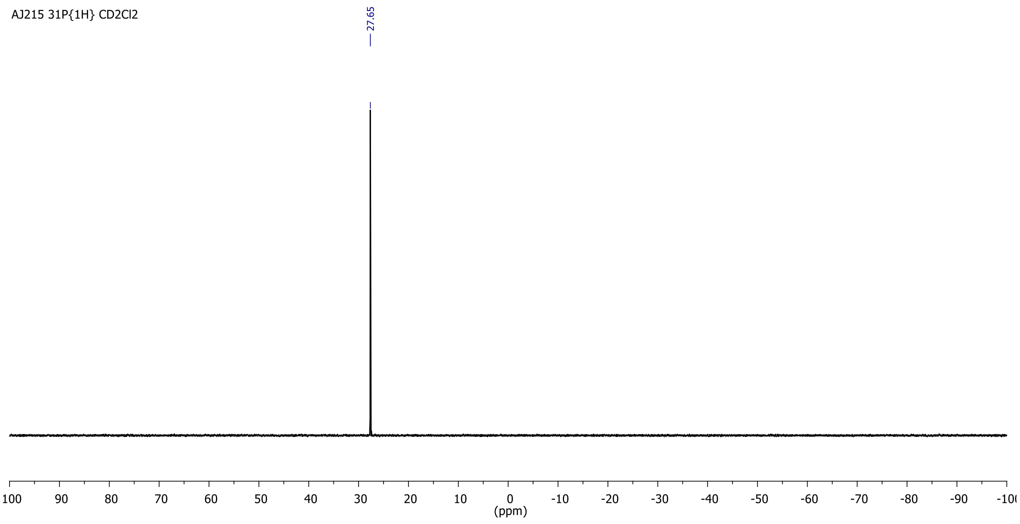

Figure S13. $^{31}P\{^{1}H\}$ NMR spectrum of **6** in CD_2Cl_2.

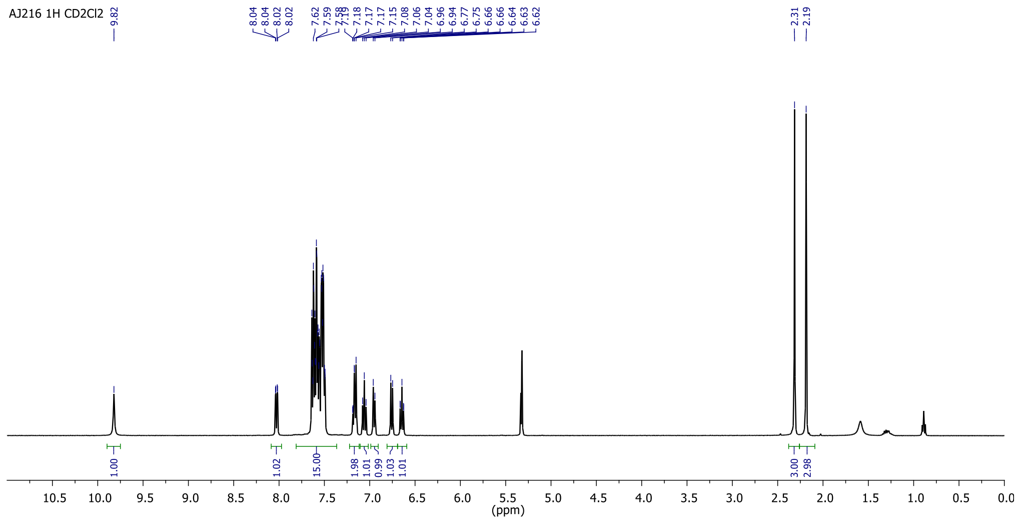

Figure S14. ^{1}H NMR spectrum of **7** in CD_2Cl_2.

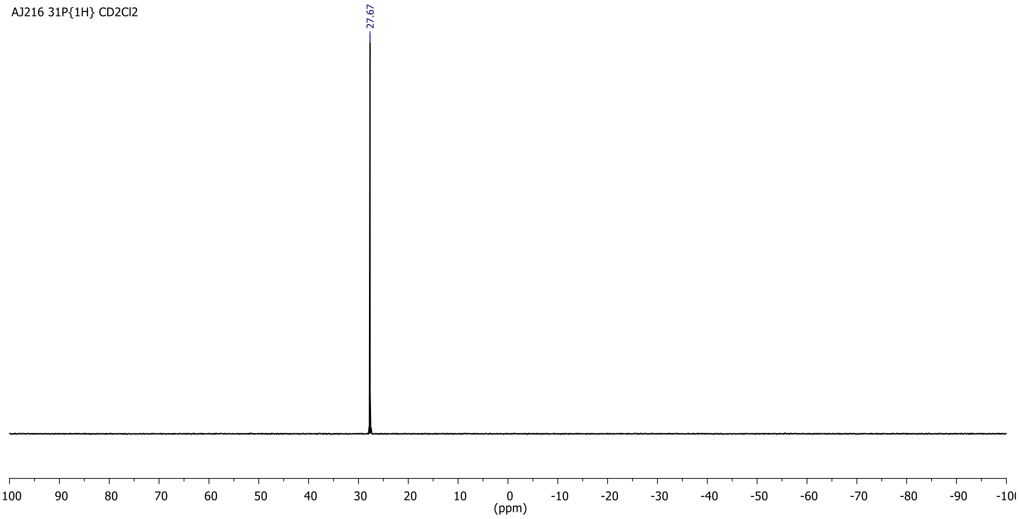

Figure S15. $^{31}P\{^{1}H\}$ NMR spectrum of **7** in CD_2Cl_2.

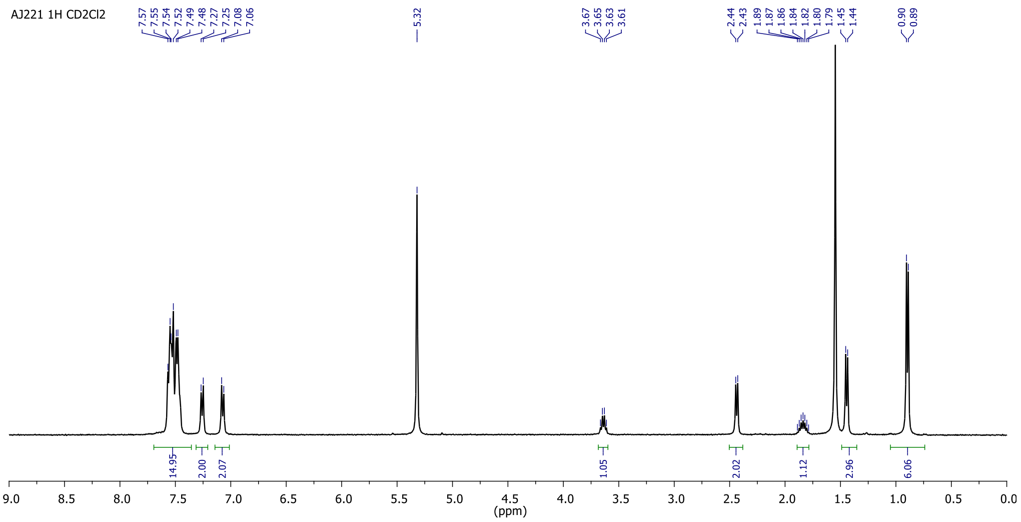

Figure S16. ^{1}H NMR spectrum of **8** in CD_2Cl_2.

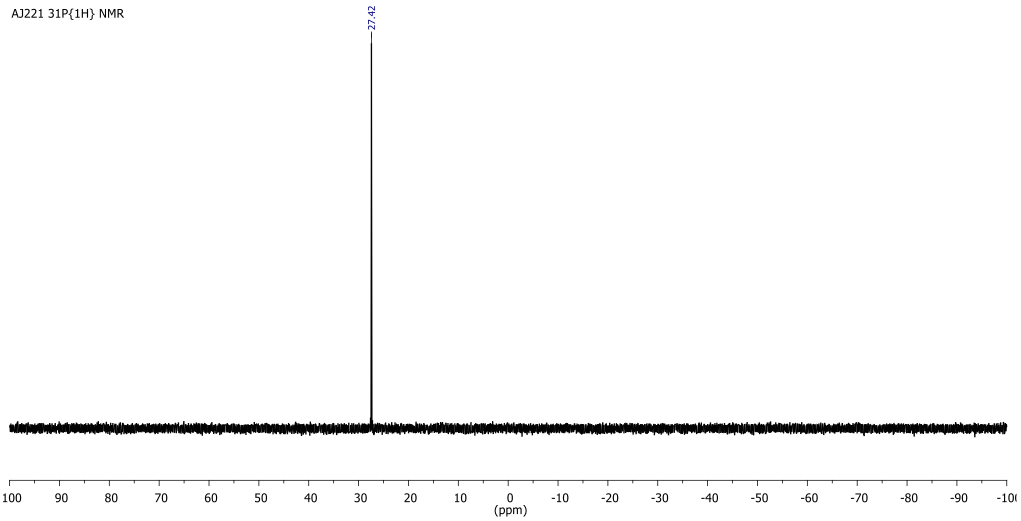

Figure S17. $^{31}P\{^{1}H\}$ NMR spectrum of **8** in CD_2Cl_2.

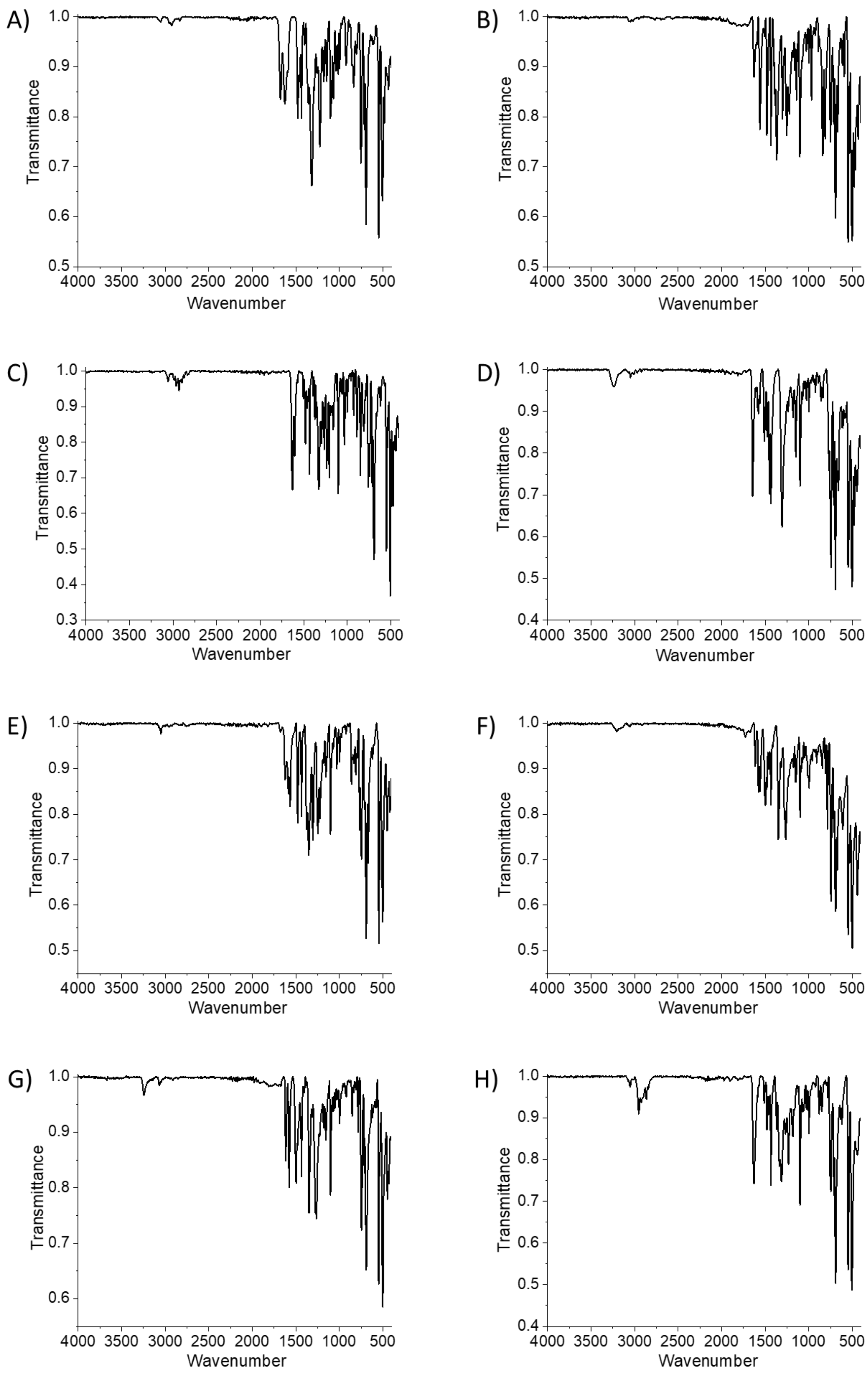

Figure S18. ATR-FTIR spectra of **1-8** (A-H) in the solid form.

Table S1. Crystallographic data for complexes **1-4**.

**Metal complex**	**1**	**2**	**3**	**4**
CCDC No.	2175741	2175743	2175742	2175738
formula	$C_{37}H_{30}AuClNO_4P$	$C_{31}H_{22}AuF_2O_3P$	$C_{32}H_{28}AuO_3P$	$C_{32}H_{25}AuCl_2NO_2P$
Fw	816.01	708.42	688.48	754.37
Crystal system	monoclinic	monoclinic	monoclinic	orthorhombic
Space group	P2(1)/c	P2(1)/n	P2(1)	Pbca
a, Å	16.005(6)	11.632(3)	13.2107(4)	11.146(2)
b, Å	12.135(4)	11.016(3)	12.0368(4)	24.058(5)
c, Å	16.626(6)	20.856(5)	17.3384(6)	42.262(8)
α, deg.	90	90	90	90
β, deg.	99.248(6)	100.801(5)	96.519(2)	90
γ, deg.	90	90	90	90
V, Å^3	3187.2(19)	2625.0(11)	2739.23(16)	11333(4)
Z	4	4	4	16
D_{calcd}, Mg/m^3	1.701	1.793	1.669	1.769
2θ / deg.	2.09 to 26.00	1.87 to 26.00	5.13 to 144.452	1.69 to 26.00
Reflections collected	24071	19994	20914	84598
Independent reflections	6246	5156	9307	11132
Goodness-of-fit on F^2	0.985	0.923	1.055	1.072
R_1, wR_2 [$I \geq 2\sigma(I)$]	0.0426, 0.0911	0.0520, 0.0943	0.0486, 0.1243	0.0619, 0.0949
R_1, wR_2 [all data]	0.0578, 0.0953	0.0811, 0.1035	0.0526, 0.1273	0.1027, 0.1032

Table S2. Crystallographic data for complexes **5-8**.

**Metal complex**	**5**	**6**	**7**	**8**
CCDC No.	2175737	2175736	2175739	2175740
formula	$C_{25}H_{20}AuO_3P$	$C_{32}H_{26}AuClNO_2P$	$C_{33}H_{29}AuNO_2P$	$C_{31}H_{32}AuO_2P$
Fw	596.35	719.92	699.51	664.50
Crystal system	monoclinic	triclinic	triclinic	monoclinic
Space group	P2(1)/n	P-1	P-1	P2(1)
a, Å	13.545(3)	8.8587(16)	8.6860(18)	13.325(5)
b, Å	11.373(3)	10.6137(19)	10.558(2)	12.768(5)
c, Å	27.757(6)	14.364(3)	15.503(3)	16.802(7)
α, deg.	90	90.750(3)	80.834(3)	90
β, deg.	100.052(4)	90.402(3)	74.403(4)	102.997(7)
γ, deg.	90	96.359(3)	89.873(4)	90
V, Å^3	4210.0(16)	1342.1(4)	1350.7(5)	2785.3(19)
Z	8	2	2	4
D_{calcd}, Mg/m^3	1.882	1.781	1.720	1.585
2θ / deg.	1.49 to 26.00	1.93 to 26.99	1.96 to 26.00	1.24 to 26.00
Reflections collected	31989	11355	10565	21714
Independent reflections	8262	5752	5230	10774
Goodness-of-fit on F^2	0.964	0.944	0.967	1.010
R_1, wR_2 [$I \geq 2\sigma(I)$]	0.0395, 0.0737	0.0427, 0.0802	0.0385, 0.0770	0.0739, 0.1661
R_1, wR_2 [all data]	0.0580, 0.0773	0.0535, 0.0827	0.0459, 0.0790	0.0896, 0.1742

Table S3. Selected bond lengths (Å) and angles (°) for complexes **1-8**.

Au(I)-NSAID complex	Au-P (Å)	Au-O (Å)	P-Au-O (°)
**1**	2.2112(16)	2.063(4)	173.61(12)
**2**	2.208(2)	2.049(5)	173.20(17)
**3**	2.210(3)/ 2.208(3)	2.079(8)/ 2.040(10)	176.5(3)/ 173.4(3)
**4**	2.215(3)/ 2.216(2)	2.044(6)/ 2.050(6)	174.06(18)/ 172.94(18)
**5**	2.2095(17)/ 2.2059(17)	2.075(4)/ 2.055(4)	174.87(14)/ 176.37(13)
**6**	2.2135(16)	2.056(4)	173.40(12)
**7**	2.2131(14)	2.042(4)	176.62(12)
**8**	2.220(4)/ 2.221(4)	2.086(11)/ 2.056(10)	177.7(3)/ 177.7(3)

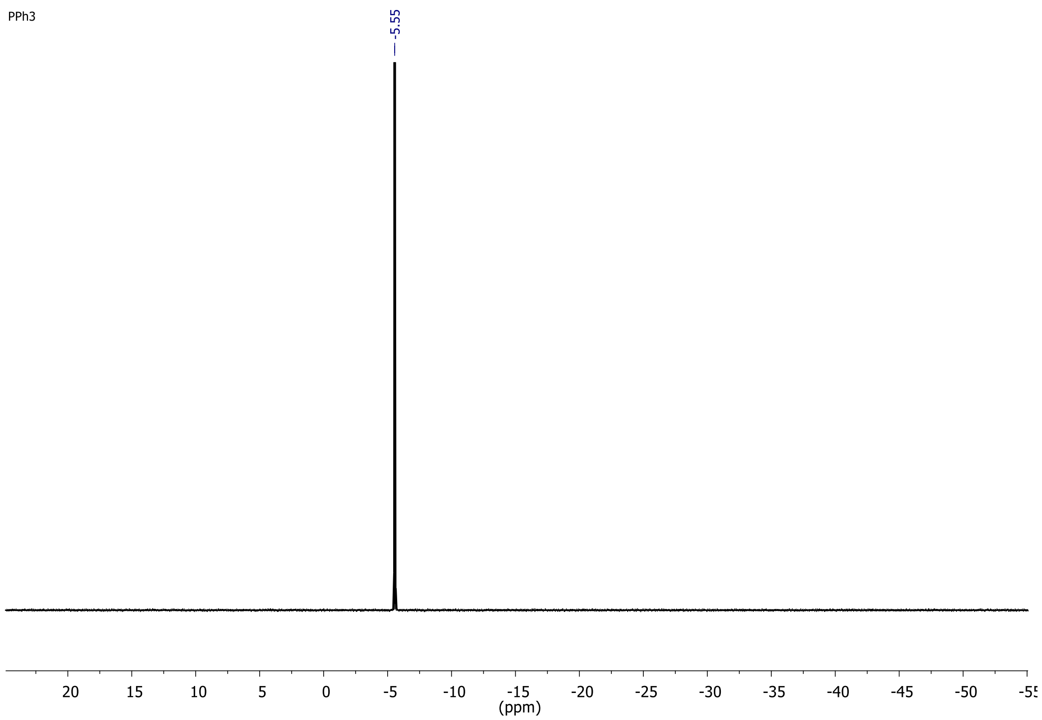

Figure S19. $^{31}P\{^{1}H\}$ NMR spectrum of free triphenylphosphine in CD_2Cl_2.

Table S4. Experimentally determined LogP values for Au(I)-NSAID complexes **1-8**.

Au(I)-NSAID complex	LogP value
**1**	1.64 ± 0.42
**2**	1.54 ± 0.44
**3**	0.71 ± 0.04
**4**	1.08 ± 0.09
**5**	0.75 ± 0.01
**6**	1.17 ± 0.37
**7**	0.83 ± 0.23
**8**	1.19 ± 0.01

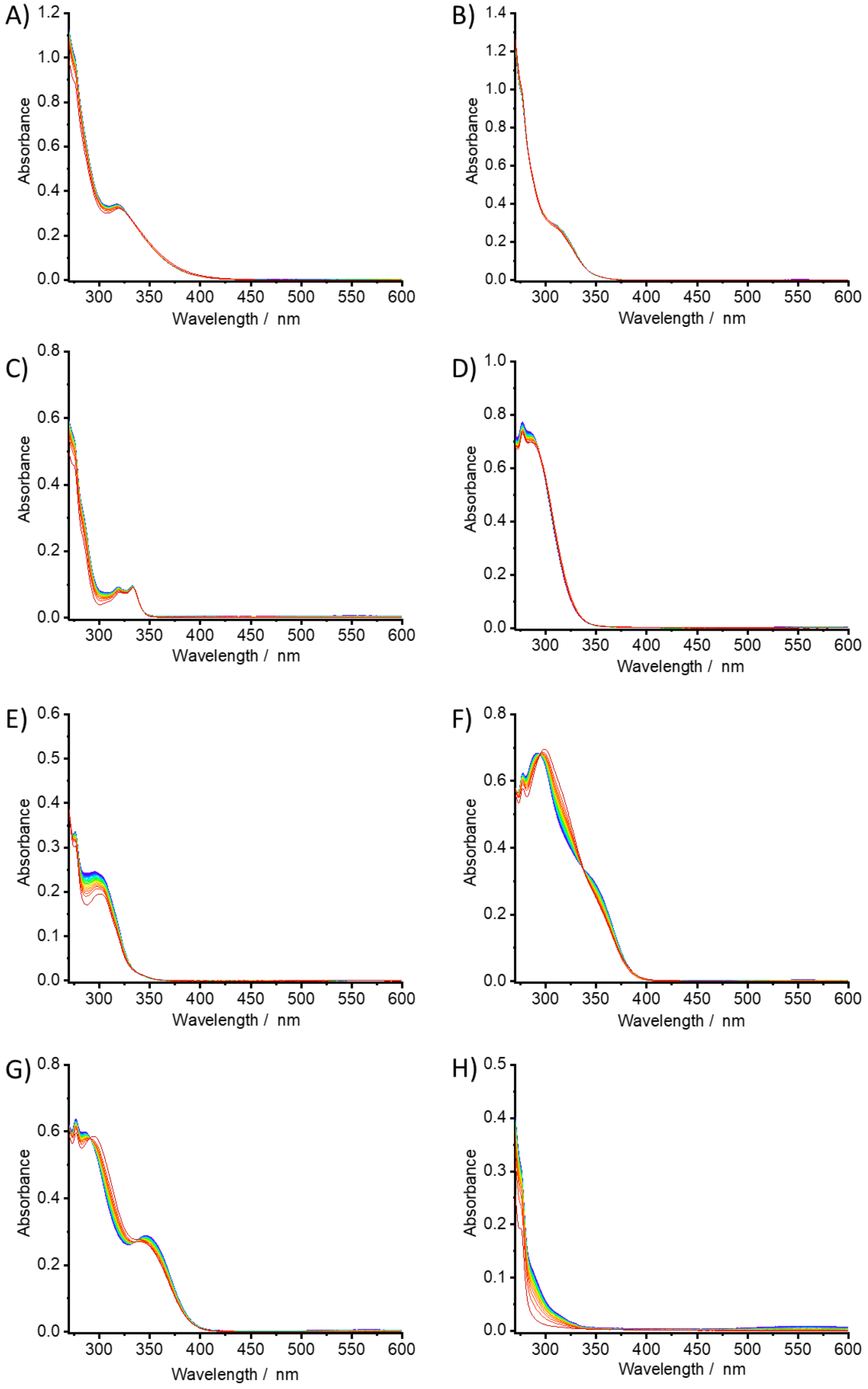

Figure S20. UV-Vis spectra of **1-8** (A-H) (all 50 μM) in DMSO over the course of 24 h (recorded at 1 h intervals) at 37 °C.

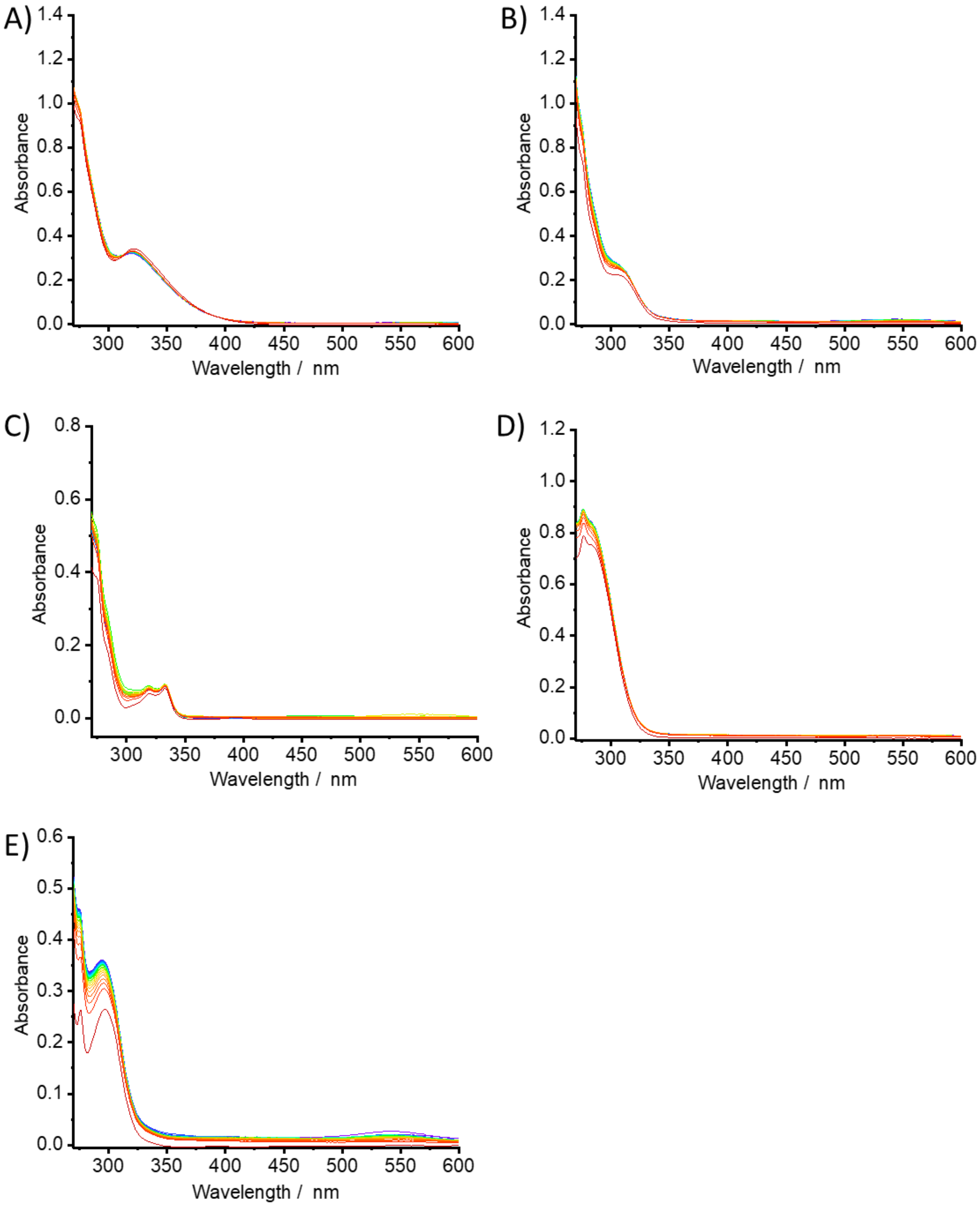

Figure S21. UV-Vis spectra of **1-5** (A-E) (all 50 μM) in PBS:DMSO (1:1) over the course of 24 h (recorded at 1 h intervals) at 37 °C.

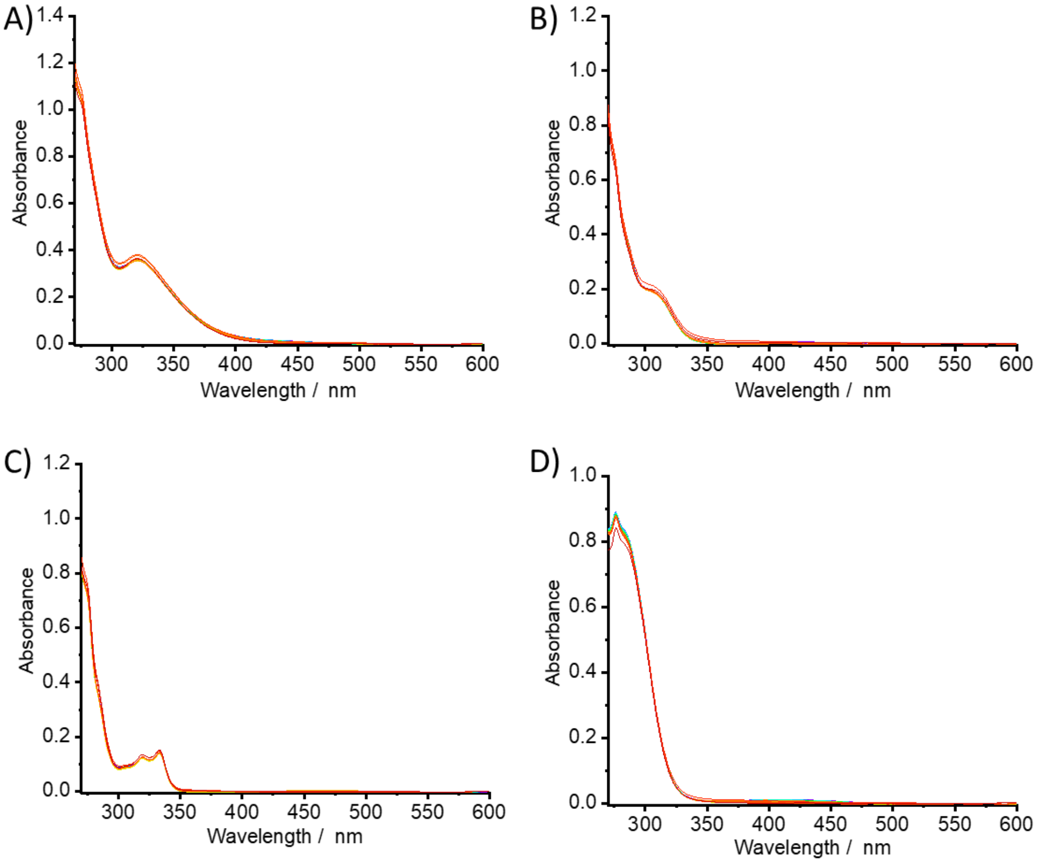

Figure S22. UV-Vis spectra of **1-4** (A-D) (all 50 μM) in MEGM:DMSO (1:1) over the course of 24 h (recorded at 1 h intervals) at 37 °C.

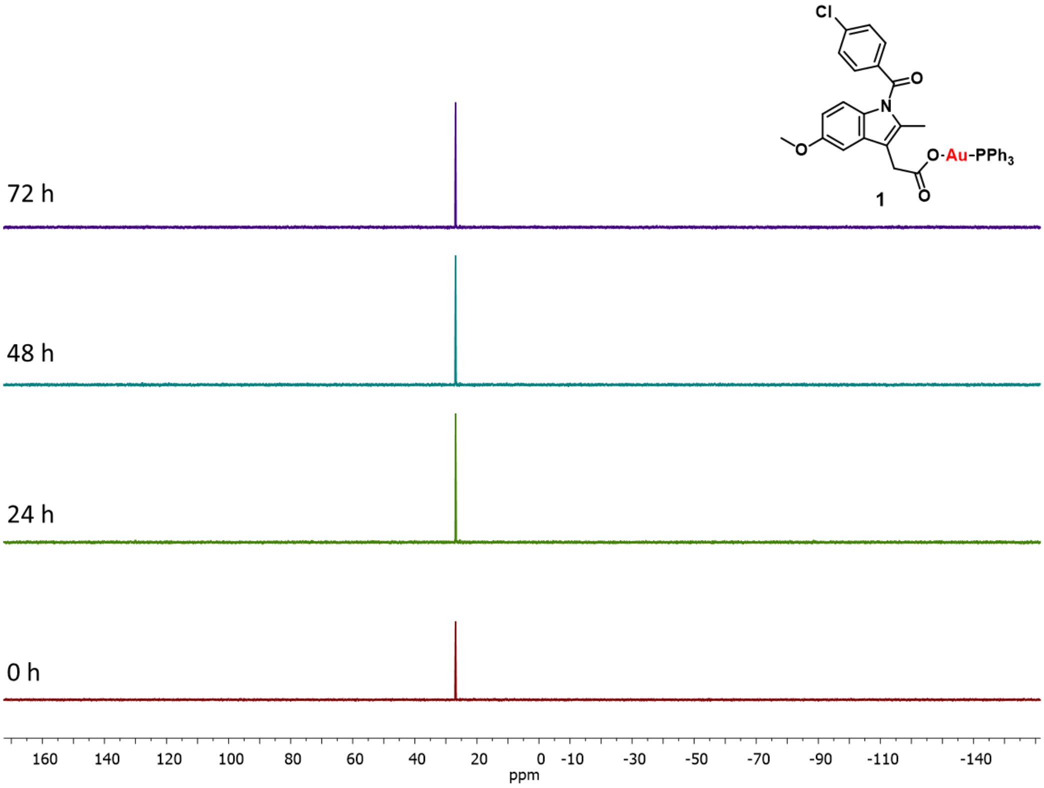

Figure S23. $^{31}P\{^{1}H\}$ NMR spectra of **1** (10 mM) in DMSO-d_6 over the course of 72 h.

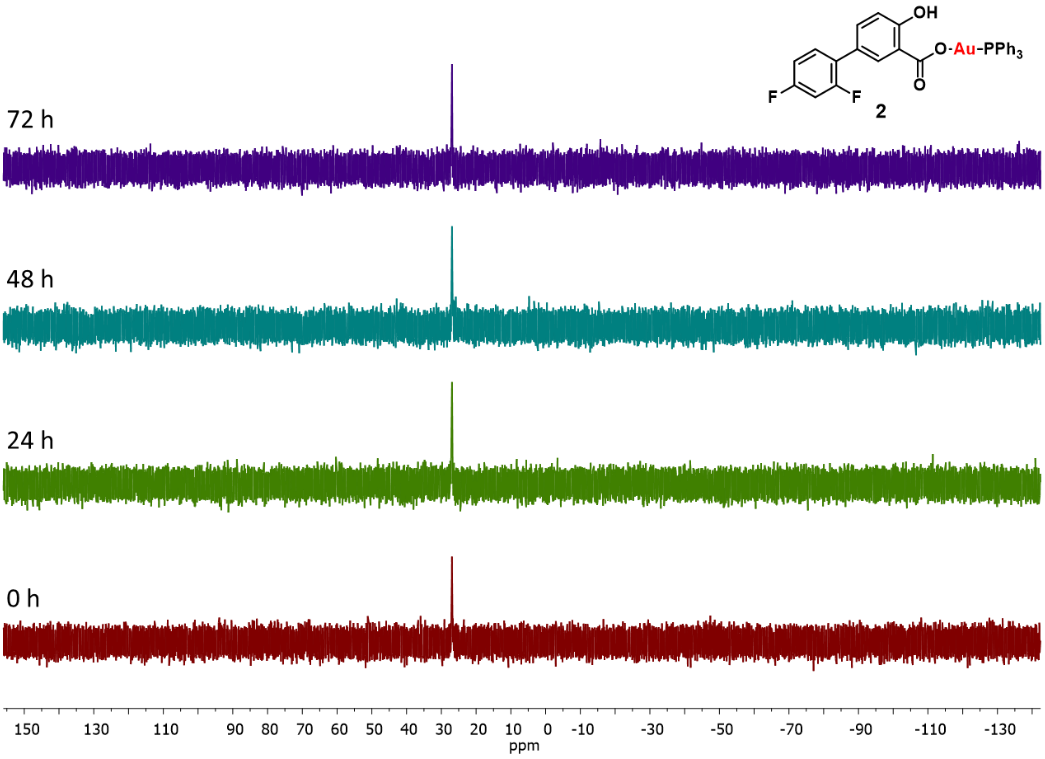

Figure S24. $^{31}P\{^{1}H\}$ NMR spectra of **2** (10 mM) in DMSO-d_6 over the course of 72 h.

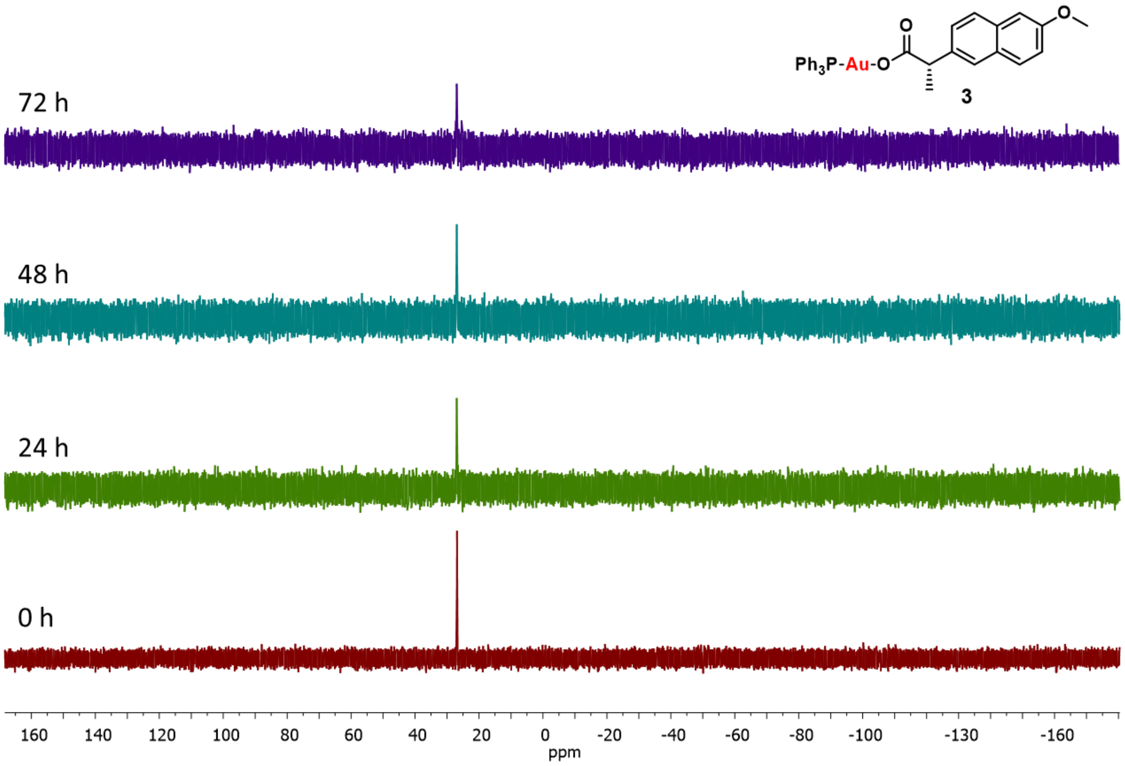

Figure S25. $^{31}P\{^{1}H\}$ NMR spectra of **3** (10 mM) in DMSO-d_6 over the course of 72 h.

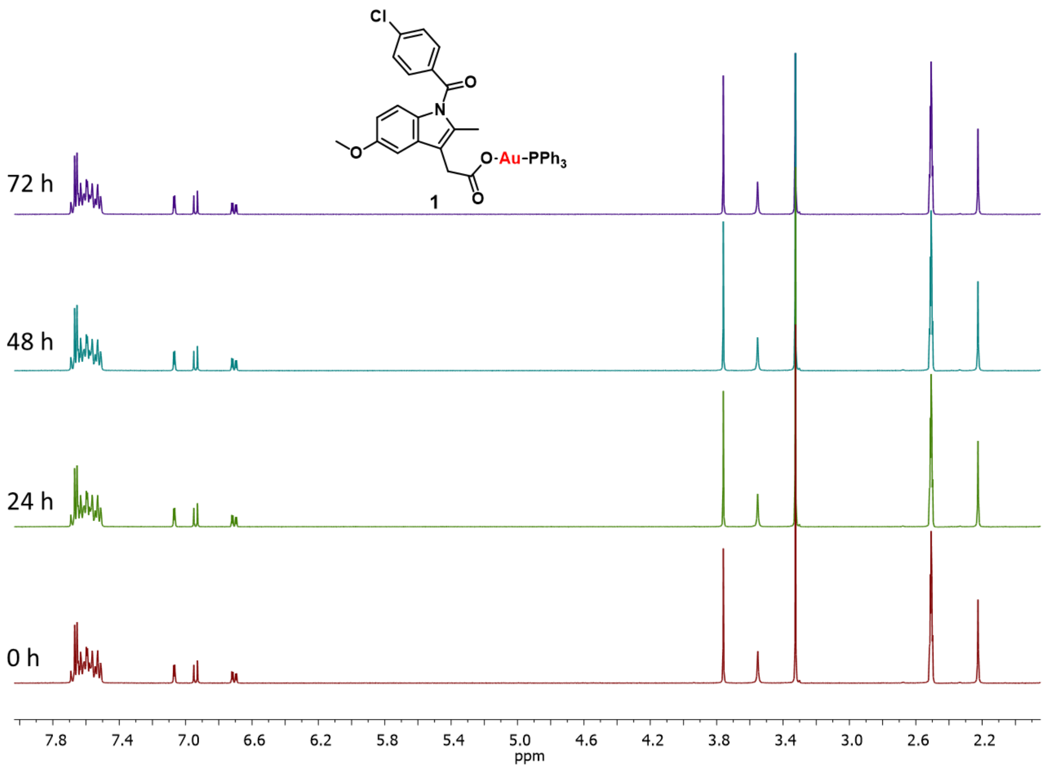

Figure S26. ^{1}H NMR spectra of **1** (10 mM) in DMSO-d_6 over the course of 72 h.

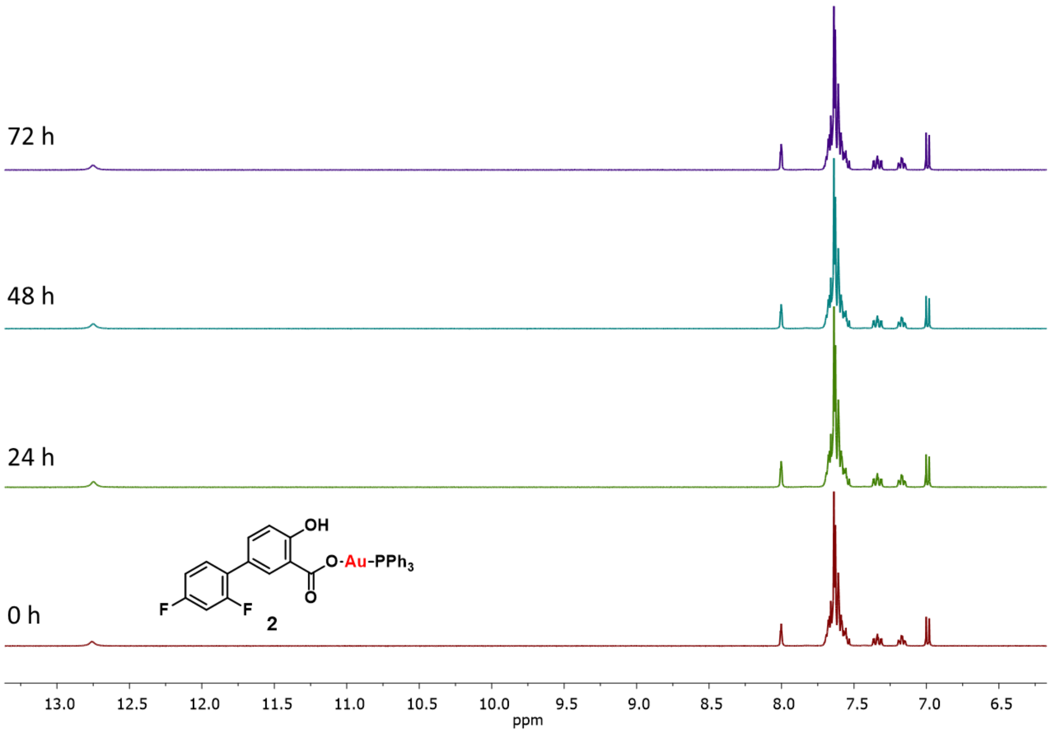

Figure S27. ^{1}H NMR spectra of **2** (10 mM) in DMSO-d_6 over the course of 72 h.

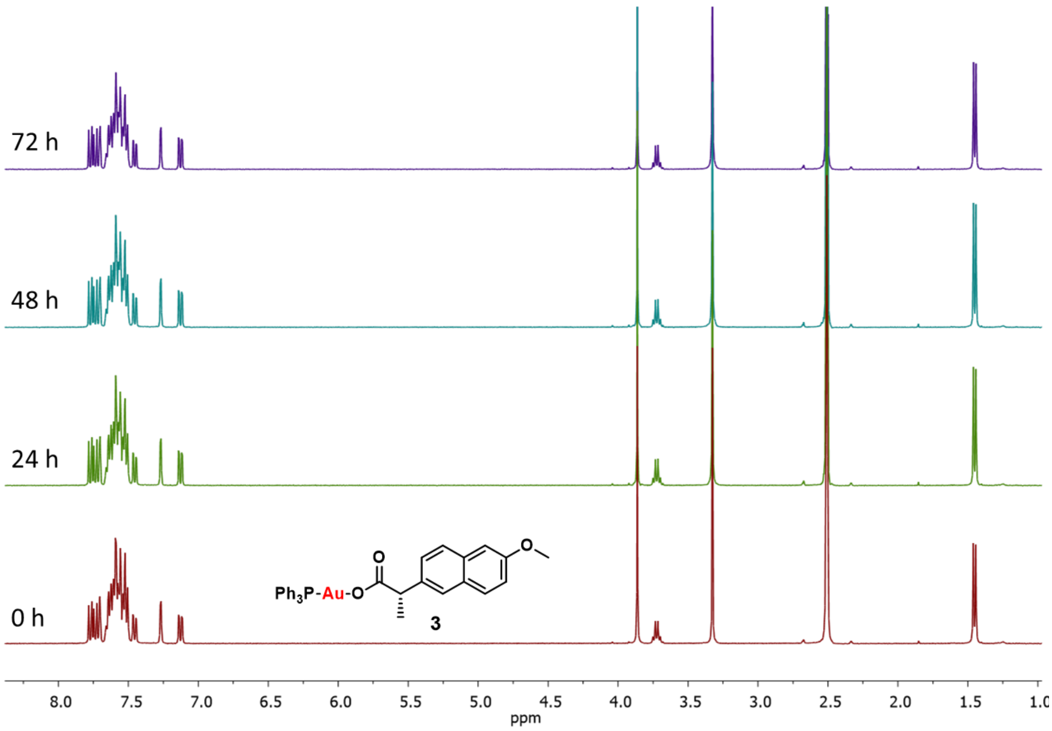

Figure S28. ^{1}H NMR spectra of **3** (10 mM) in DMSO-d_6 over the course of 72 h.

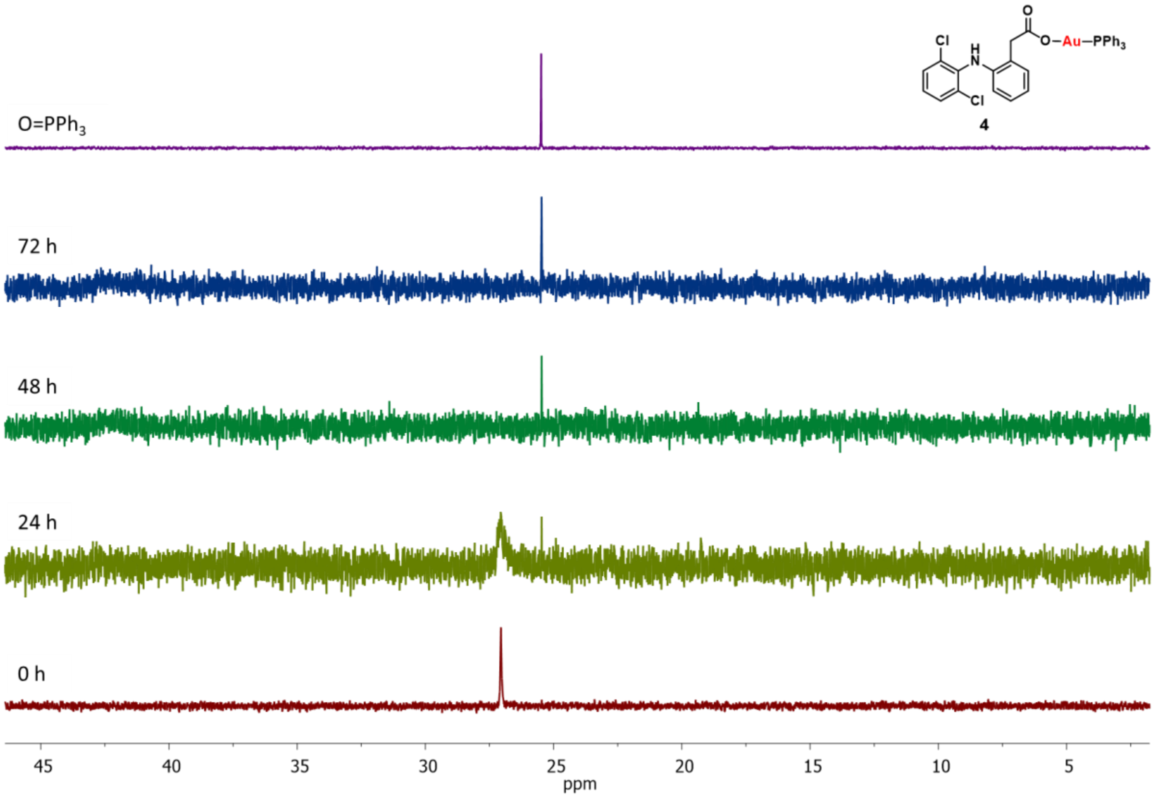

Figure S29. $^{31}P\{^{1}H\}$ NMR spectra of **4** (10 mM) in DMSO-d_6 over the course of 72 h. The $^{31}P\{^{1}H\}$ NMR spectrum of O=PPh_3 (10 mM) in DMSO-d_6 is also provided.

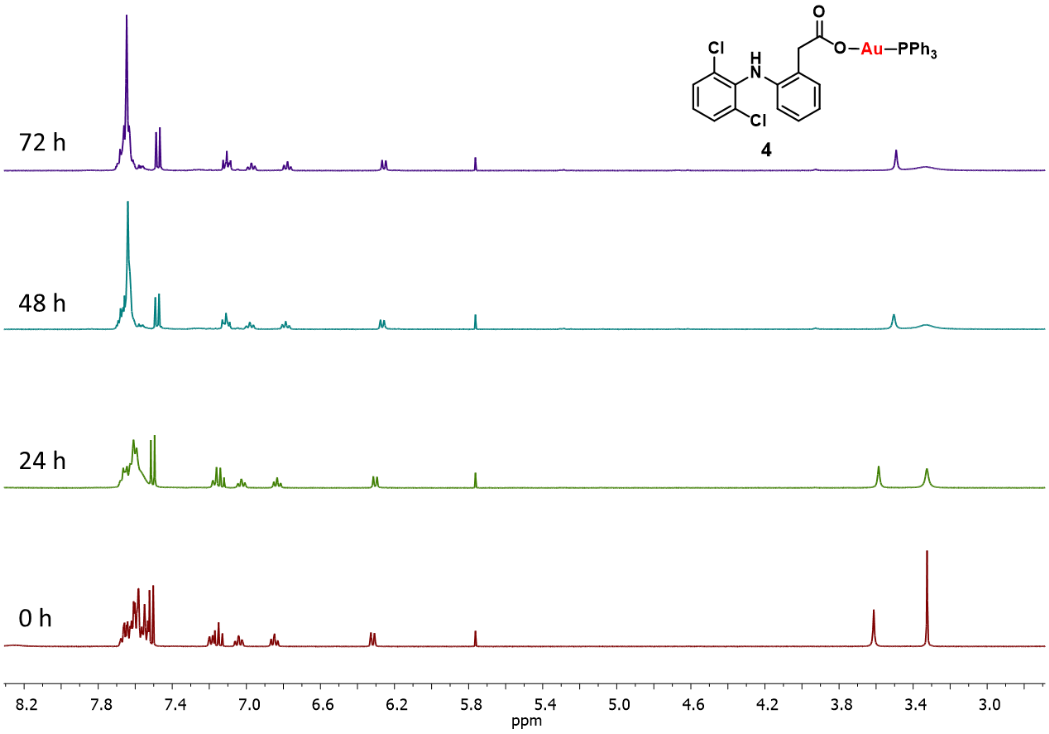

Figure S30. ^{1}H NMR spectra of **4** (10 mM) in DMSO-d_6 over the course of 72 h.

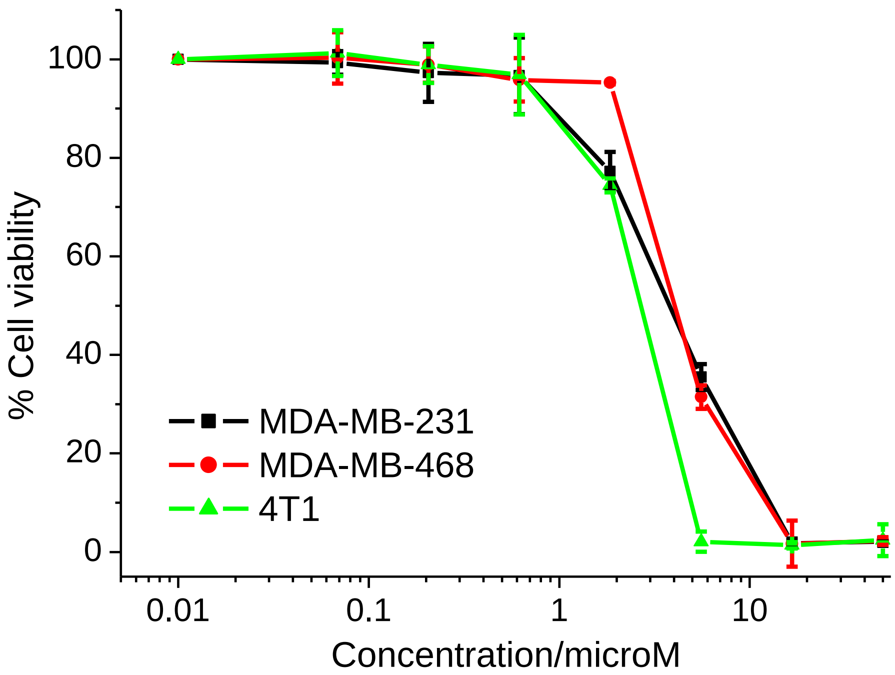

Figure S31. Representative dose-response curves for the treatment of MDA-MB-231, MDA-MB-468, and 4T1 cells with **1** after 72 h incubation.

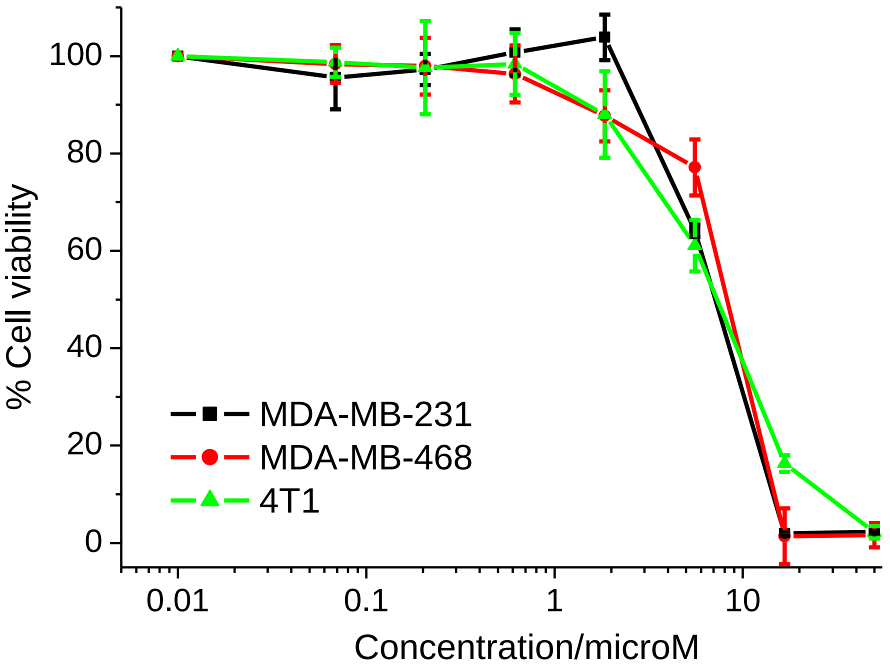

Figure S32. Representative dose-response curves for the treatment of MDA-MB-231, MDA-MB-468, and 4T1 cells with **2** after 72 h incubation.

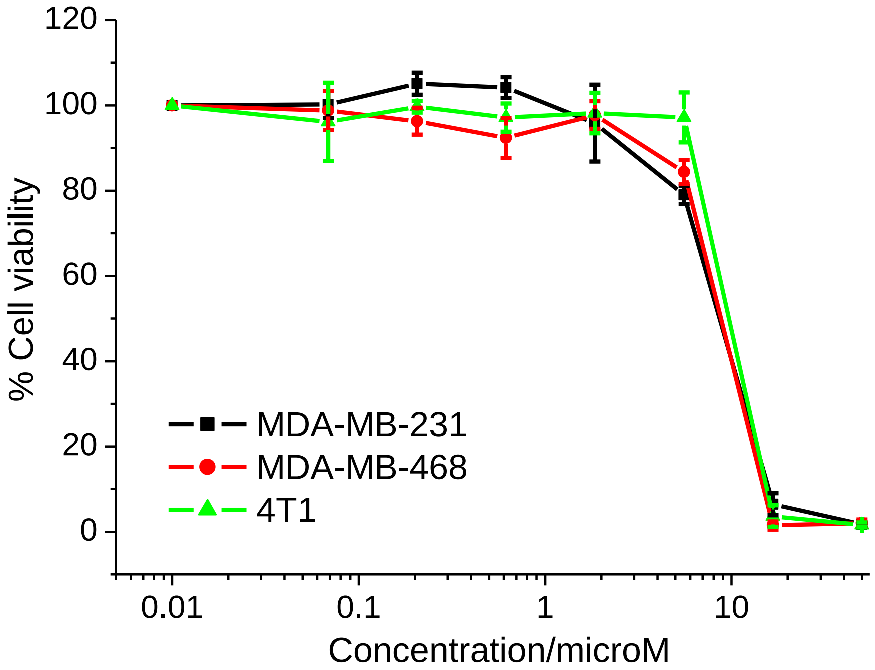

Figure S33. Representative dose-response curves for the treatment of MDA-MB-231, MDA-MB-468, and 4T1 cells with **3** after 72 h incubation.

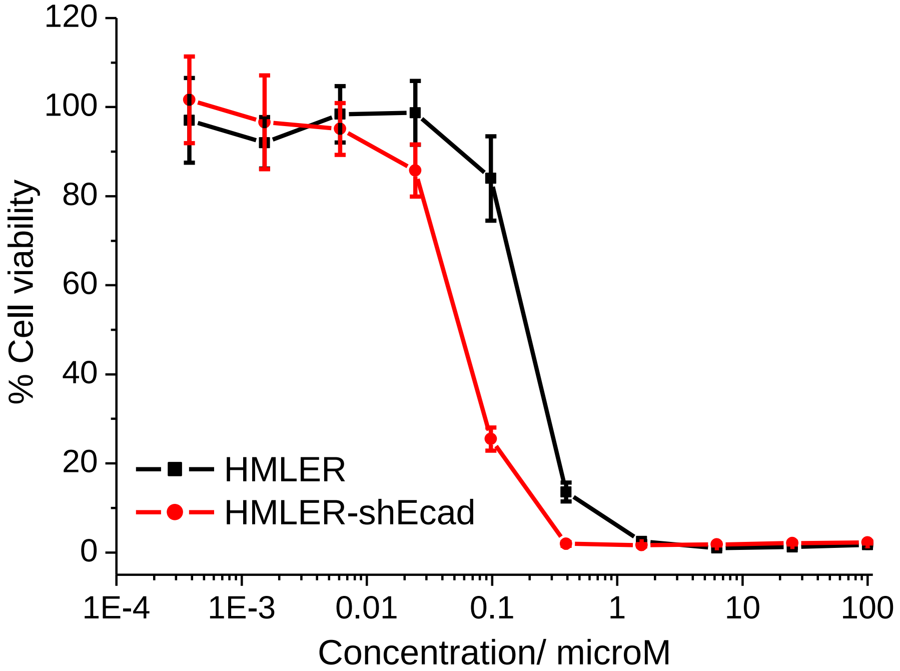

Figure S34. Representative dose-response curves for the treatment of HMLER and HMLER-shEcad cells with **1** after 72 h incubation.

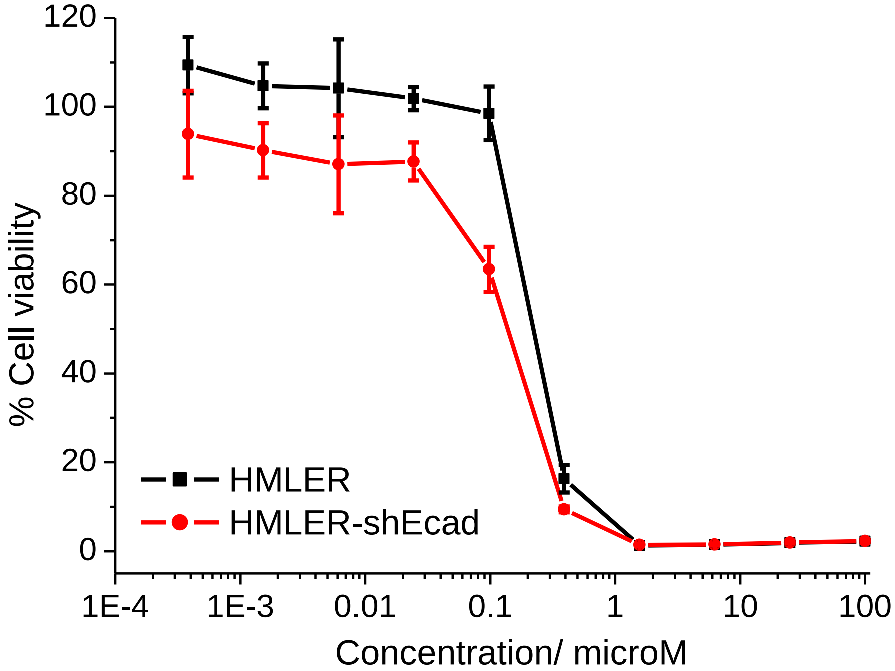

Figure S35. Representative dose-response curves for the treatment of HMLER and HMLER-shEcad cells with **2** after 72 h incubation.

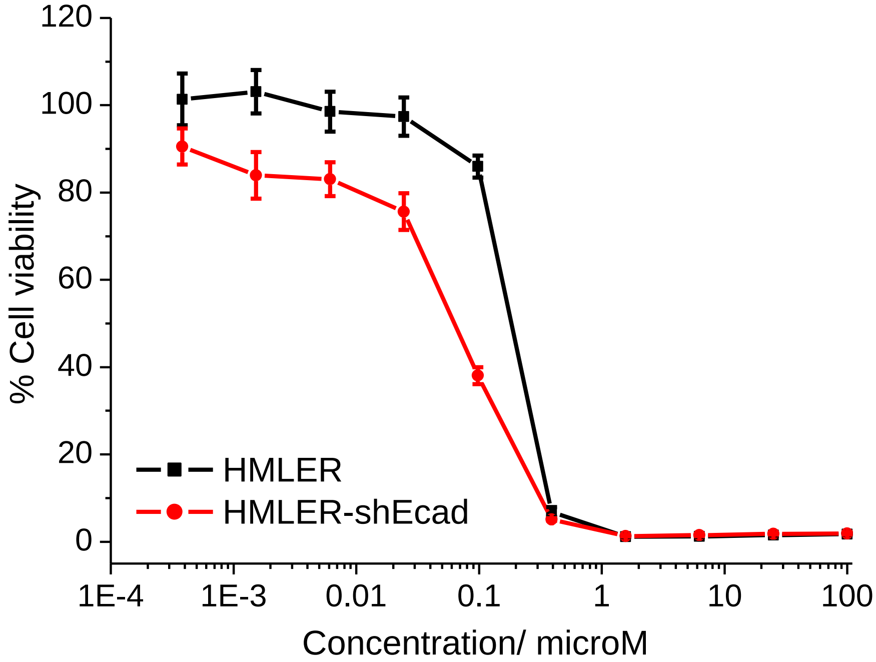

Figure S36. Representative dose-response curves for the treatment of HMLER and HMLER-shEcad cells with **3** after 72 h incubation.

Table S5. IC_{50} values of the gold(I)-NSAID complexes, **1**-**3** against MDA-MB-231, MDA-MB-468, and 4T1 cells. [a]Determined after 72 h incubation (mean of three independent experiments ± SD).

Au(I)-NSAID complex	MDA-MB-231 [μM] [a]	MDA-MB-468 [μM] [a]	4T1 [μM] [a]
**1**	3.94 ± 0.30	4.47 ± 0.17	2.24 ± 0.11
**2**	5.79 ± 0.79	7.12 ± 0.10	7.25 ± 0.4
**3**	7.77 ± 0.41	6.48 ± 1.43	10.08 ± 0.86

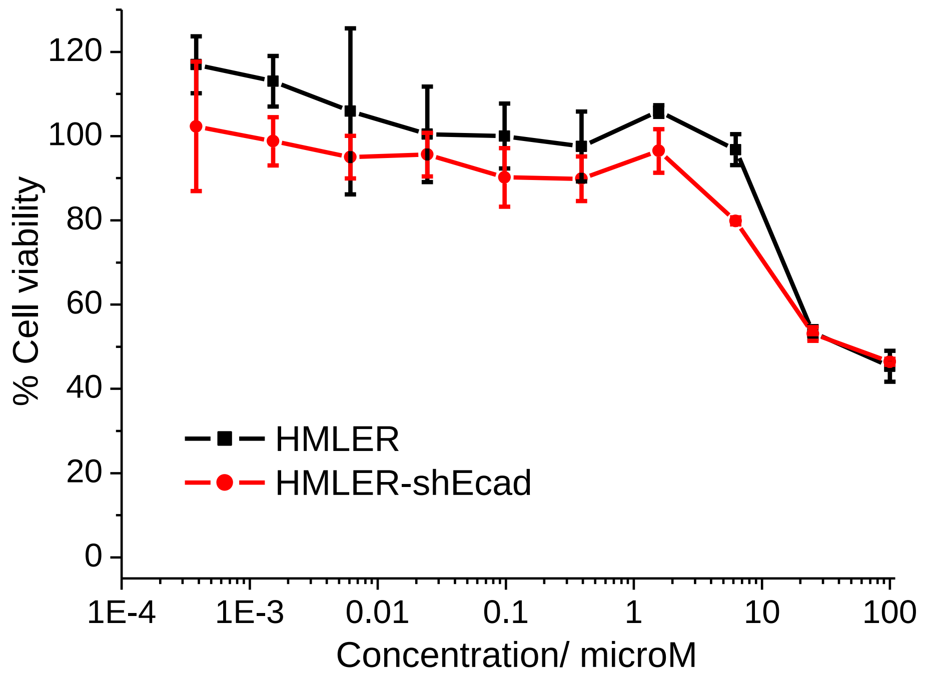

Figure S37. Representative dose-response curves for the treatment of HMLER and HMLER-shEcad cells with 5-fluorouracil after 72 h incubation.

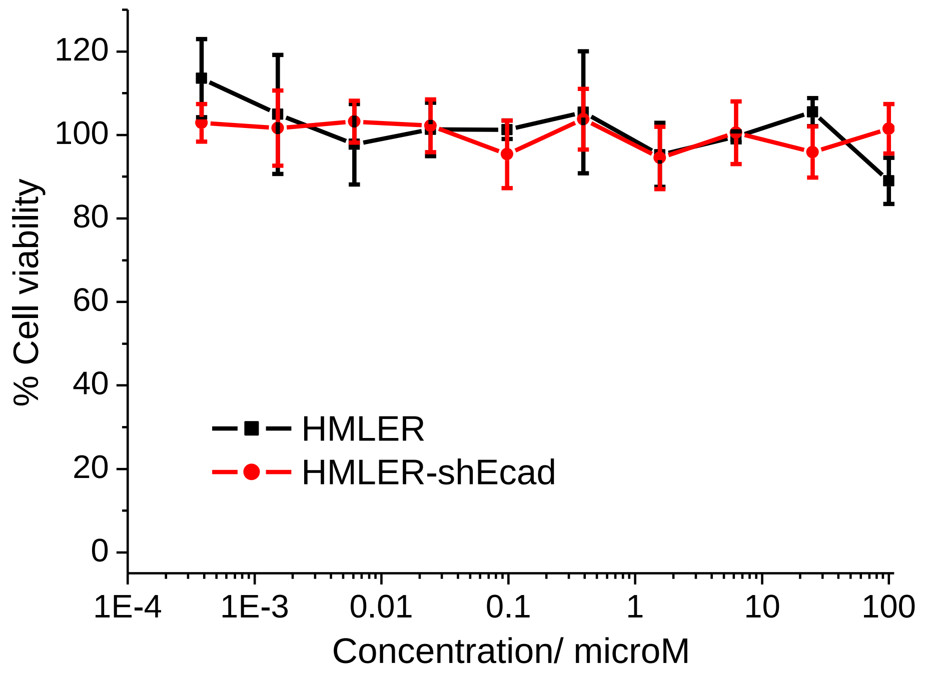

Figure S38. Representative dose-response curves for the treatment of HMLER and HMLER-shEcad cells with capecitabine after 72 h incubation.

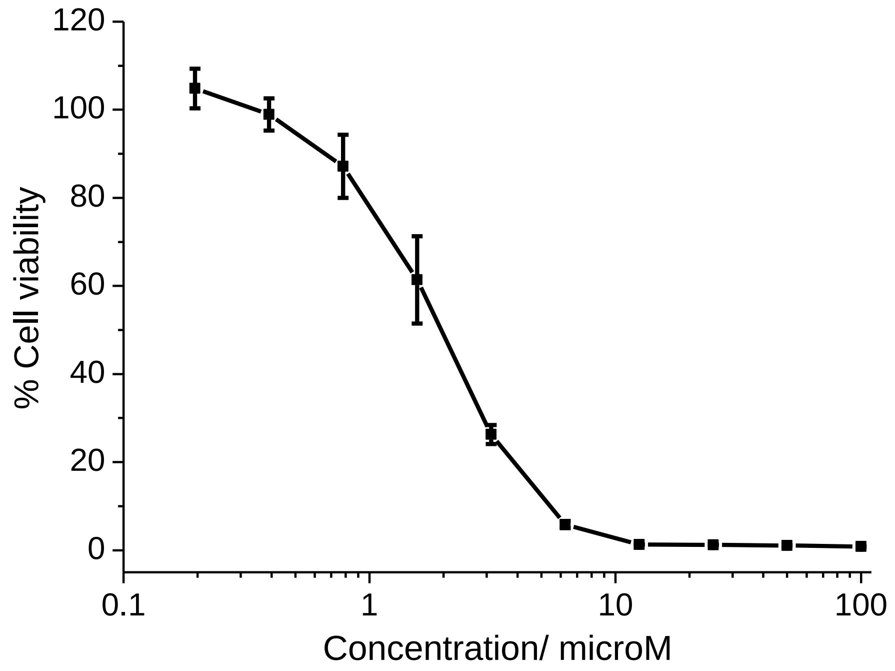

Figure S39. Representative dose-response curves for the treatment of HEK 293 cells with **1** after 72 h incubation.

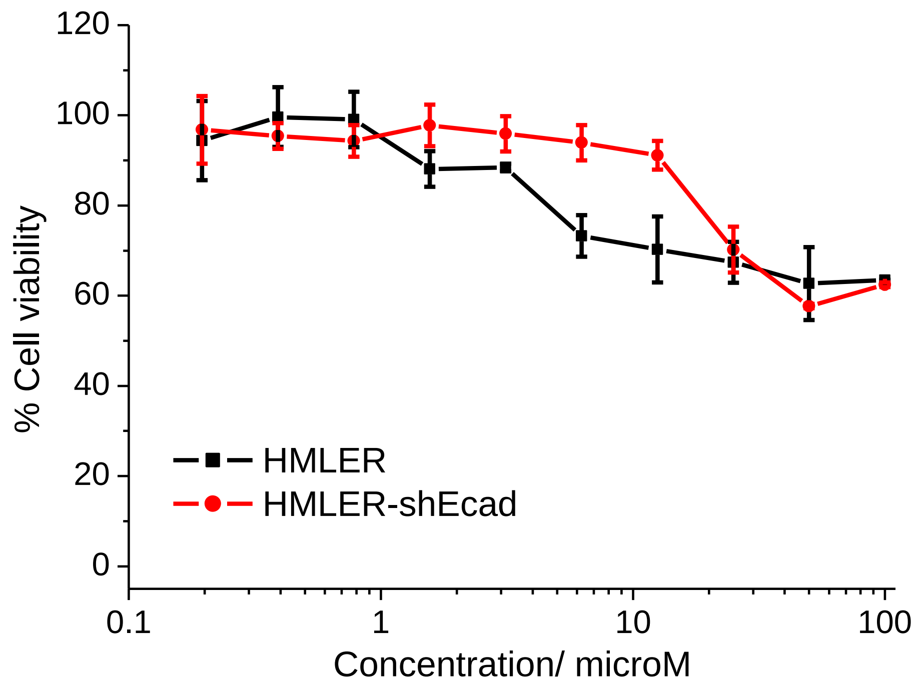

Figure S40. Representative dose-response curves for the treatment of HMLER and HMLER-shEcad cells with indomethacin after 72 h incubation.

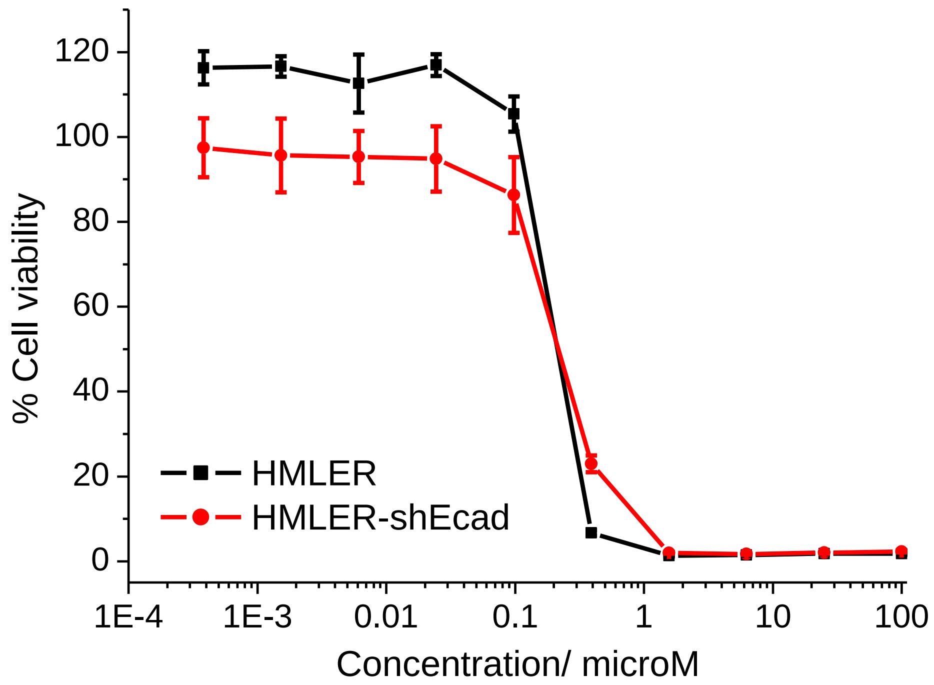

Figure S41. Representative dose-response curves for the treatment of HMLER and HMLER-shEcad cells with chloro(triphenylphosphine)gold(I) after 72 h incubation.

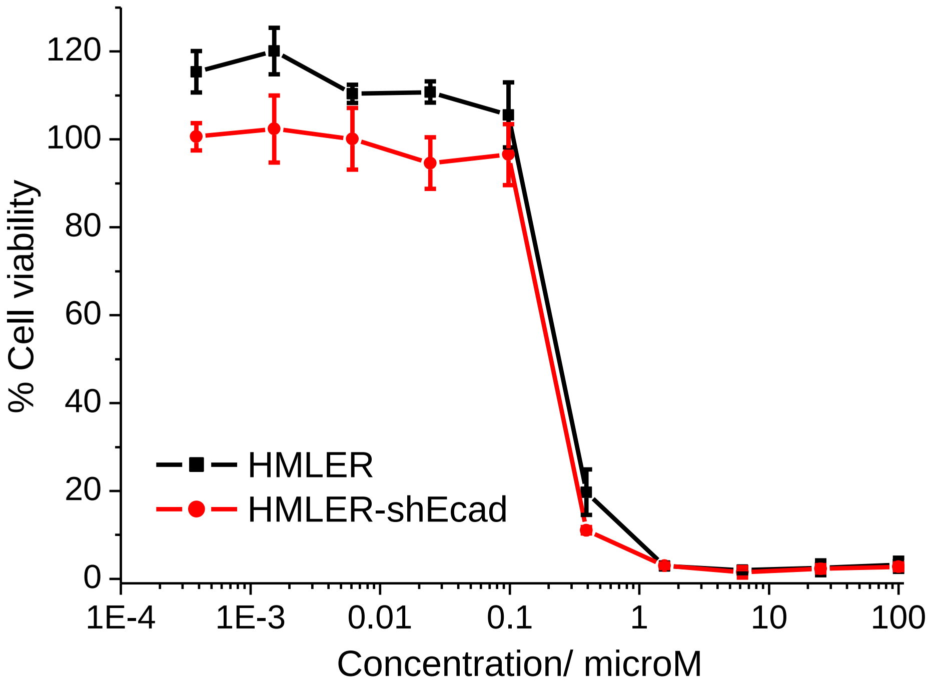

Figure S42. Representative dose-response curves for the treatment of HMLER and HMLER-shEcad cells with a 1:1 mixture of indomethacin and chloro(triphenylphosphine)gold(I) after 72 h incubation.

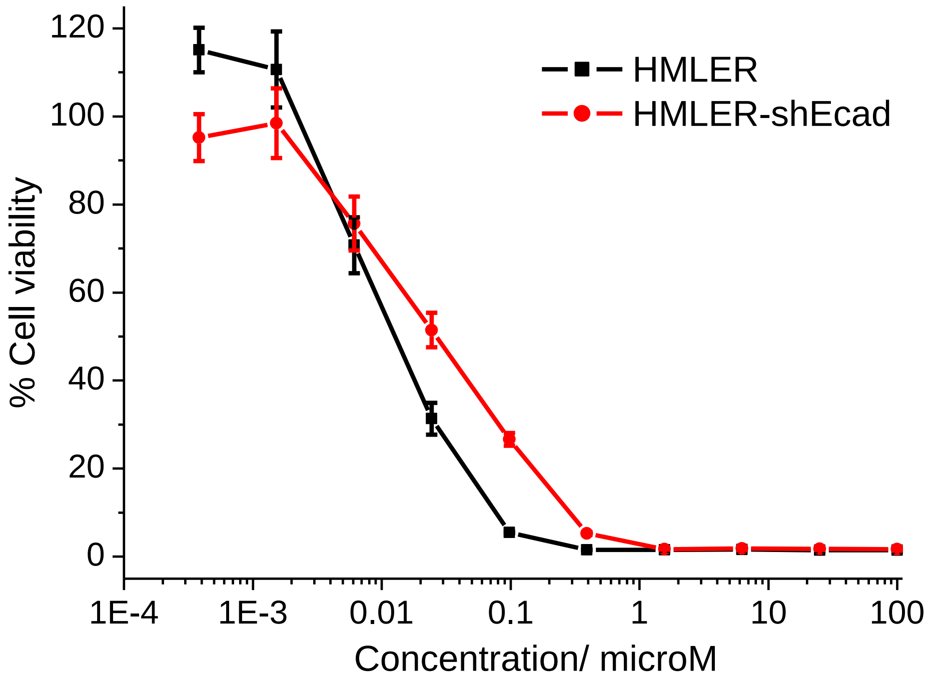

Figure S43. Representative dose-response curves for the treatment of HMLER and HMLER-shEcad cells with auranofin after 72 h incubation.

Table S6. IC_{50} values of indomethacin, chloro(triphenylphosphine)gold(I), a 1:1 mixture of indomethacin and chloro(triphenylphosphine)gold(I), and auranofin against HMLER and HMLER-shEcad cells. [a]Determined after 72 h incubation (mean of three independent experiments ± SD).

Test compounds(s)	HMLER [nM] [a]	HMLER-shEcad [nM] [a]
indomethacin	> 100000	> 100000
chloro(triphenylphosphine)gold(I)	207 ± 5	216 ± 3
indomethacin + chloro(triphenylphosphine)gold(I)	239 ± 2	208 ± 2
auranofin	13 ± 2	26 ± 5

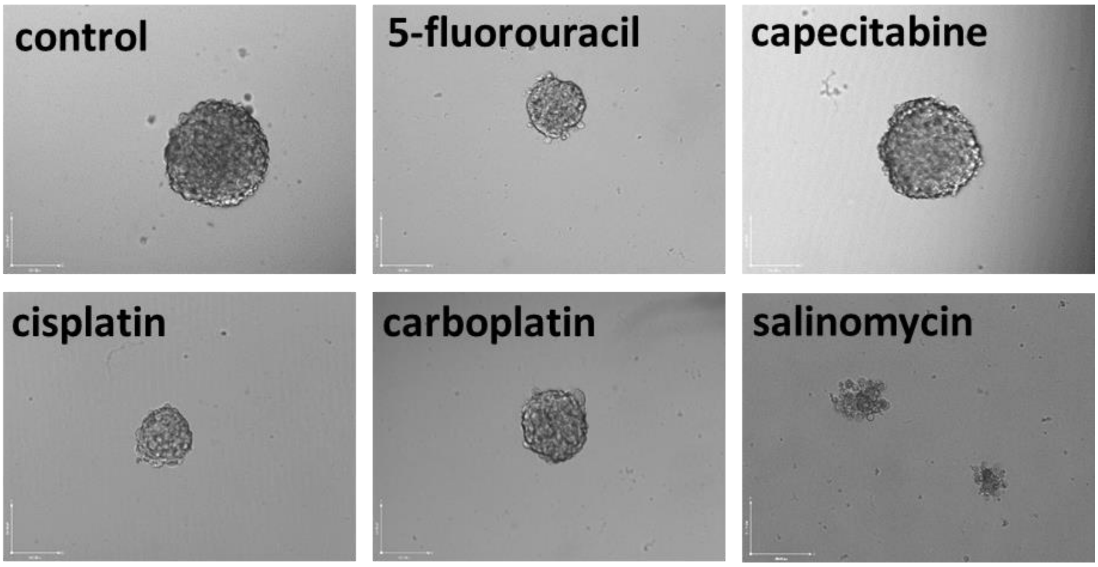

Figure S44. Representative bright-field images (x 10) of the mammospheres in the absence and presence of 5-fluorouracil, capecitabine, cisplatin, carboplatin, and salinomycin at their respective IC_{20} values after 5 day incubation.

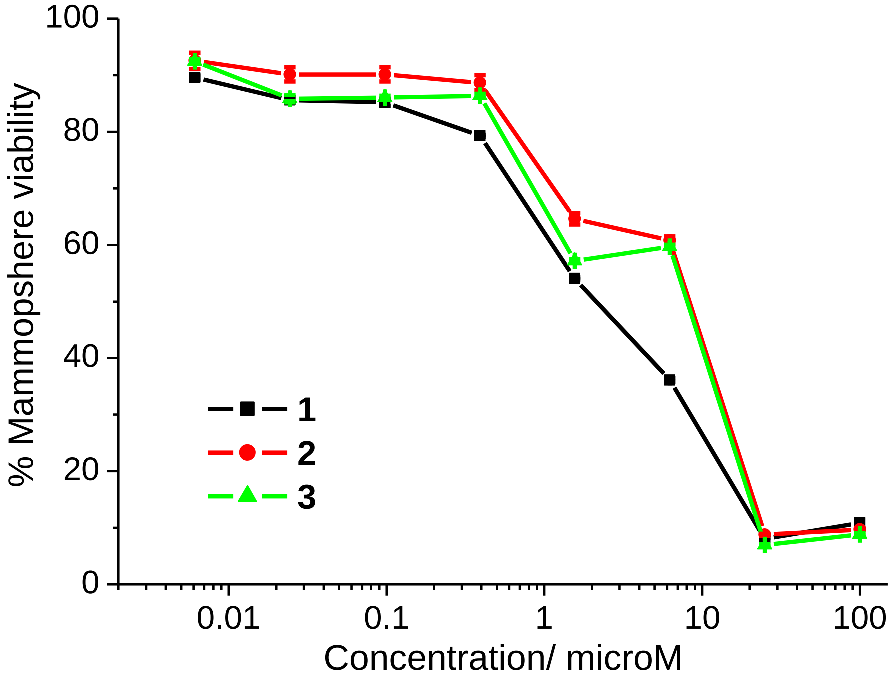

Figure S45. Representative dose-response curves for the treatment of HMLER-shEcad mammospheres with **1-3** after 5 days incubation.

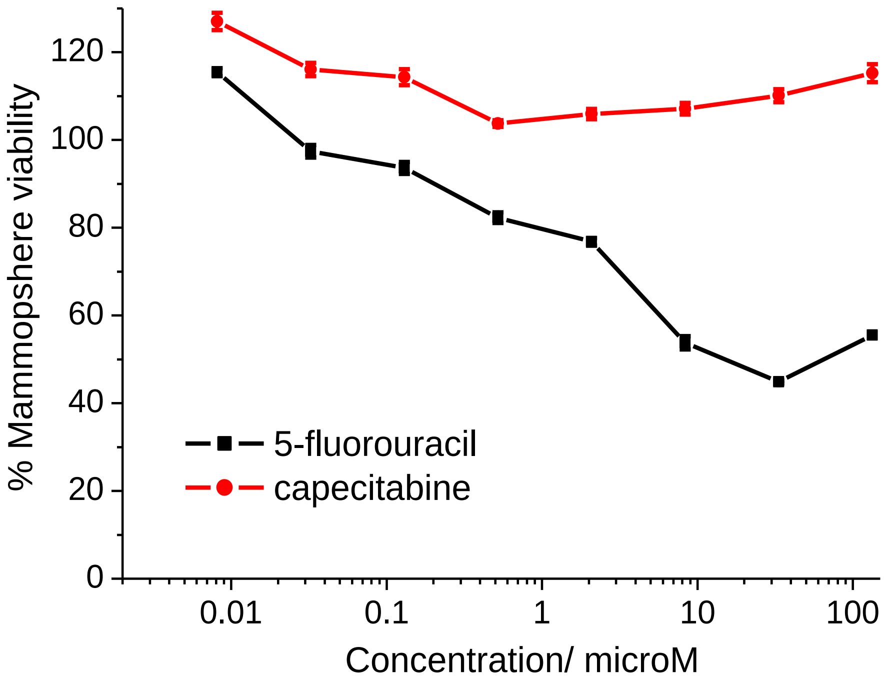

Figure S46. Representative dose-response curves for the treatment of HMLER-shEcad mammospheres with 5-fluorouracil and capecitabine after 5 days incubation.

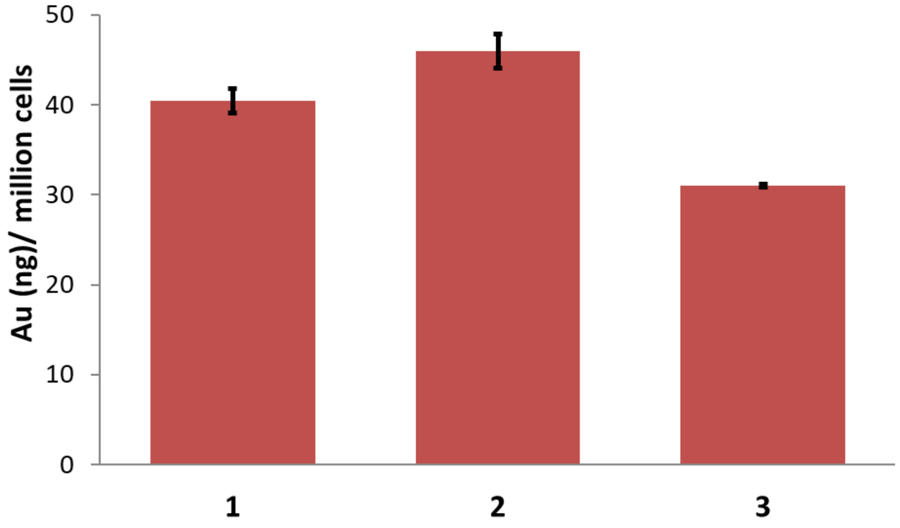

Figure S47. Gold content (ng of Au/ 10^6 cells) in HMLER-shEcad cells after treatment with **1**-**3** (0.25 µM for 24 h).

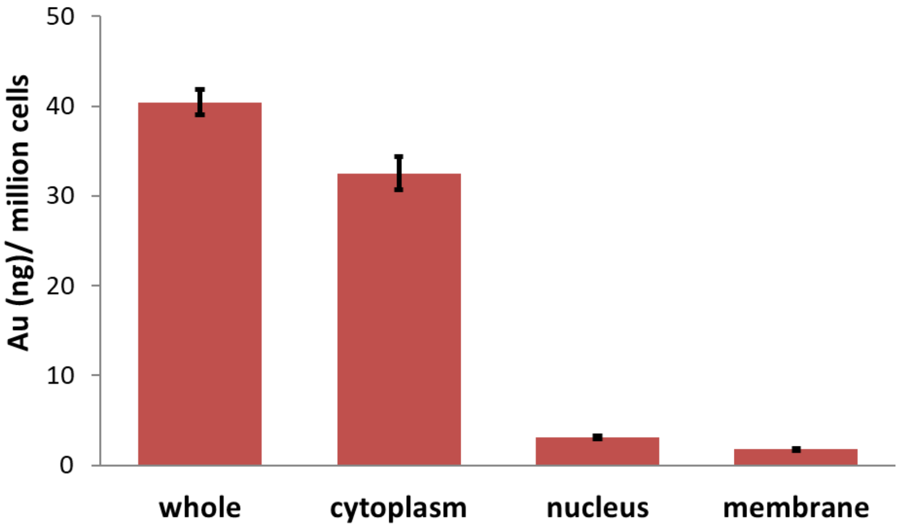

Figure S48. Gold content (ng of Au/ 10^6 cells) in various cellular components upon treatment of HMLER-shEcad cells with **1** (0.25 µM for 24 h).

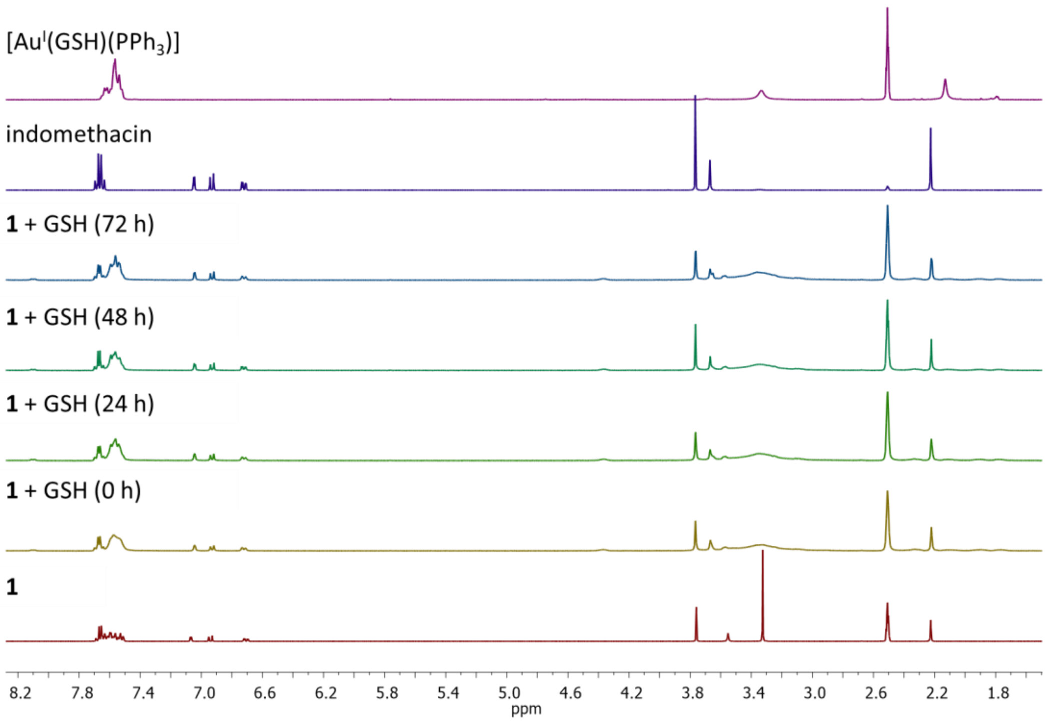

Figure S49. ^{1}H NMR spectra for complex **1** (10 mM) in DMSO-d_6, in the absence and presence of glutathione (GSH, 10 mM) over the course of 72 h at 37 °C. The ^{1}H NMR spectra of indomethacin and [Au^{I}(GSH)(PPh$_3$)] (both 10 mM) in DMSO-d_6 are also provided.

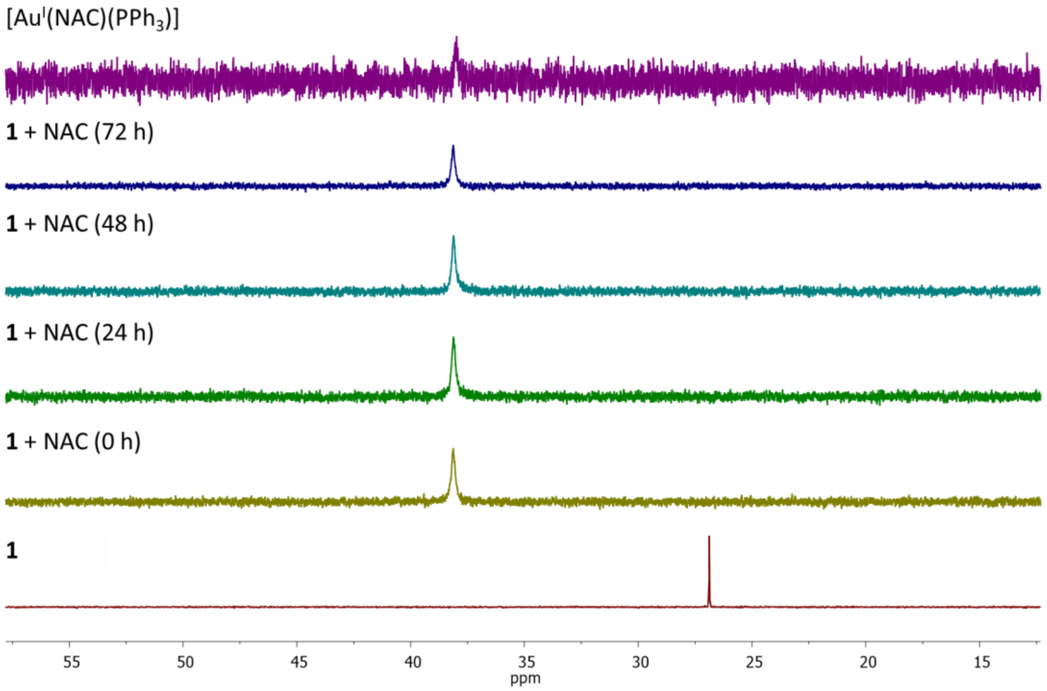

Figure S50. ^{31}P{^{1}H} NMR spectra for complex **1** (10 mM) in DMSO-d_6, in the absence and presence of *N*-acetylcysteine (NAC, 10 mM) over the course of 72 h at 37 °C. The ^{31}P{^{1}H} NMR spectrum of [Au^{I}(NAC)(PPh$_3$)] (10 mM) in DMSO-d_6 is also provided.

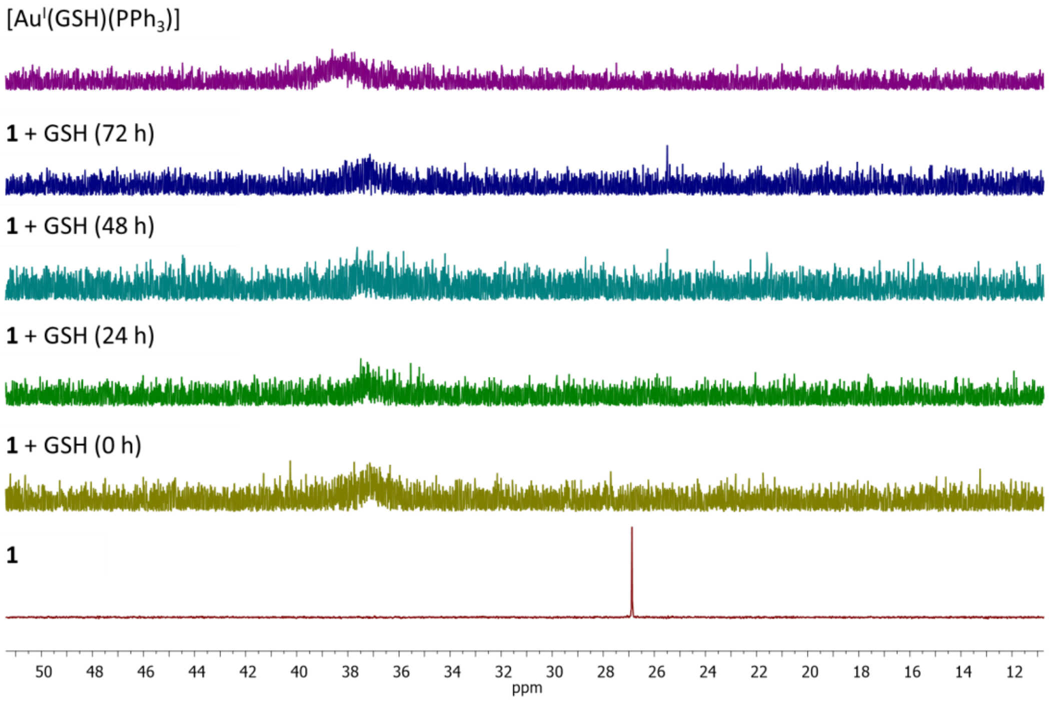

Figure S51. $^{31}P\{^1H\}$ NMR spectra for complex **1** (10 mM) in DMSO-d_6, in the absence and presence of glutathione (GSH, 10 mM) over the course of 72 h at 37 °C. The $^{31}P\{^1H\}$ NMR spectrum of [Au^{I}(GSH)(PPh$_3$)] (10 mM) in DMSO-d_6 is also provided.

Scheme S1. Representative scheme for the reaction of **1** with glutathione.

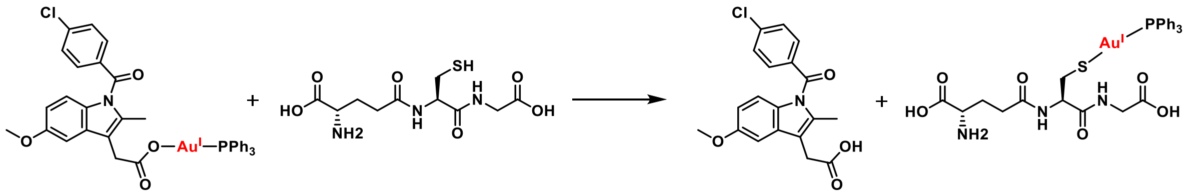

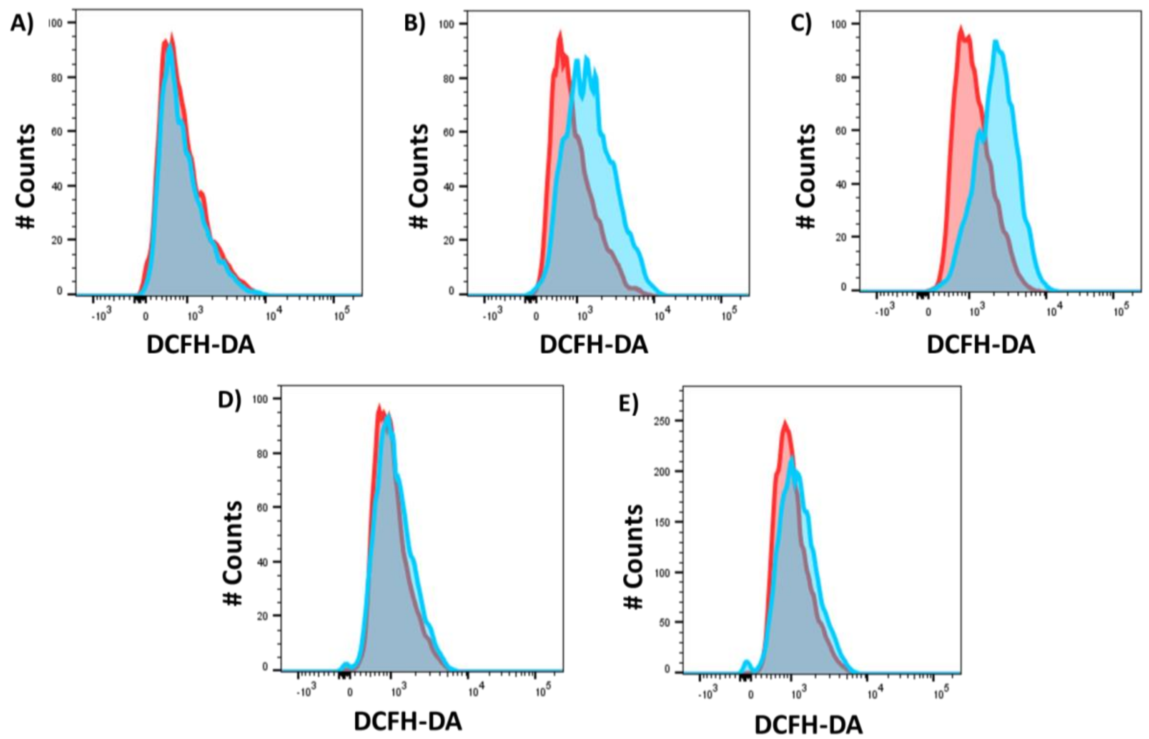

Figure S52. Representative histograms displaying the green fluorescence emitted by DCFH-DA-stained HMLER-shEcad cells (red) and HMLER-shEcad cells treated with **1** (0.4 µM) (blue) for (A) 1 h, (B) 3 h, (C) 6 h, (D) 16 h, and (E) 24 h.

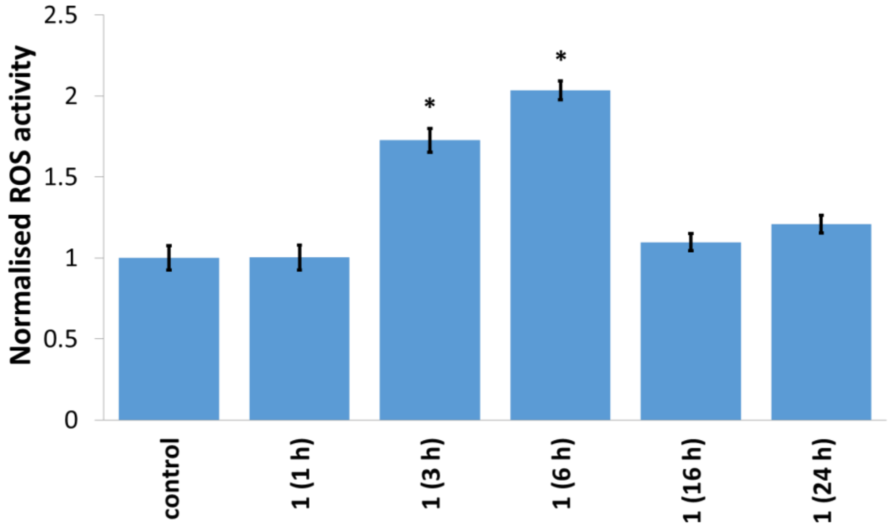

Figure S53. Summarised ROS data. Normalised ROS activity in untreated HMLER-shEcad cells (control) and HMLER-shEcad cells treated with **1** (0.4 µM for 1, 3, 6, 16, and 24 h).

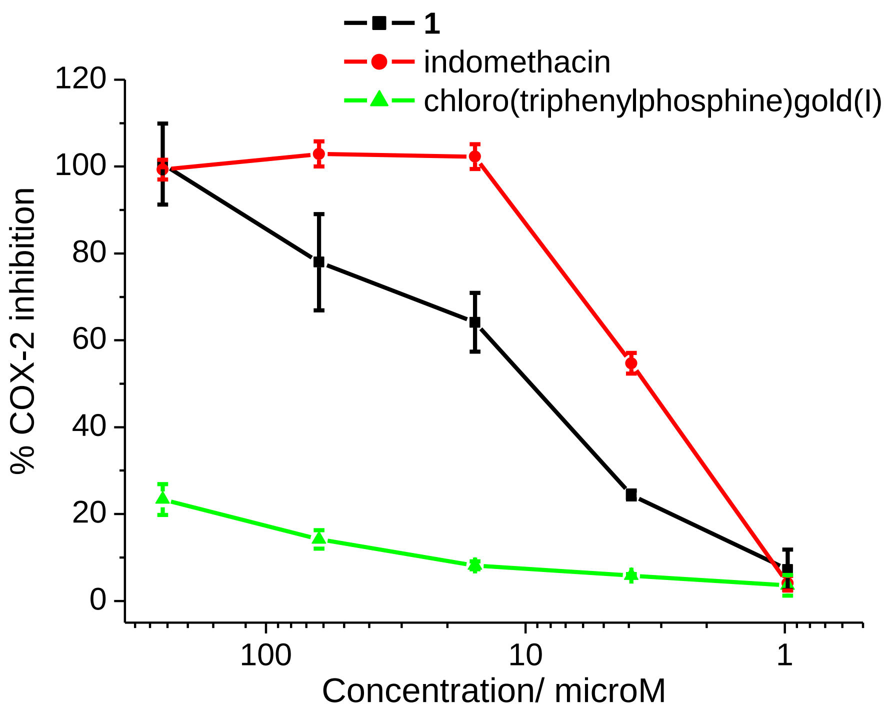

Figure S54. Representative dose-response curves for the inhibition of COX-2 activity by **1**, indomethacin, or chloro(triphenylphosphine)gold(I).

Table S7. IC_{50} values of **1**, indomethacin, and chloro(triphenylphosphine)gold(I) associated to their ability to inhibit COX-2 activity.

Compound	COX-2<br>IC_{50} value [μM]
**1**	9.44 ± 0.51
indomethacin	3.54 ± 0.23
chloro(triphenylphosphine)gold(I)	> 250

References

1. G. Sheldrick, *Acta Cryst.* , 2008, **A64**, 112-122.
